# Laser Desorption-Rapid Evaporative Ionization Mass Spectrometry (LD-REIMS): A New Tool for the High-Throughput Metabolomic and Lipidomic Profiling of Live Cells

**DOI:** 10.1021/acs.analchem.5c04847

**Published:** 2025-11-27

**Authors:** Stefania Maneta-Stavrakaki, Aurelien Tripp, Daniel Simon, Yuchen Xiang, Adrienn Molnar, Athanasios Tsalikis, Efstathios Andreas Elia, Josephine Bunch, Julia Balog, George Poulogiannis, Zoltan Takats

**Affiliations:** † Department of Metabolism, Digestion, and Reproduction, 4615 Imperial College London, Hammersmith Campus, Du Cane Rd, W12 0NN London, U.K.; ‡ Signalling and Cancer Metabolism Team, Division of Cell and Molecular Biology, 5053 The Institute of Cancer Research, 237 Fulham Rd., SW3 6JB London, U.K.; § Hevesy György PhD School of Chemistry, ELTE Eötvös Loránd University, H-1117 Budapest, Hungary; ∥ National Centre of Excellence in Mass Spectrometry Imaging (NiCE-MSI), 9917 National Physical Laboratory, Teddington, TW11 0LW London, U.K.; ⊥ Immerse Cambridge, 301 Binney St Suite 102, Cambridge, Massachusetts 02142, United States

## Abstract

Understanding the dynamic cellular metabolism is essential for gaining deeper insights into inter- and intracellular functions. In recent years, mass spectrometry (MS) has become the technology of choice for the biochemical characterization and profiling of cell lines, particularly when coupled with separation techniques such as liquid chromatography (LC-MS). However, these methods typically involve extensive sample preparation with potent organic solvents, which is labor-intensive, time-consuming, and incompatible with direct analysis of intact, live cells. Here, we propose the use of the ambient ionization technique Laser Desorption-Rapid Evaporative Ionization Mass Spectrometry (LD-REIMS) incorporated in an automated platform, for the high-throughput profiling of live or frozen cell monolayers, with minimal pretreatment. Validation experiments using 10 breast and colorectal cancer cell lines confirmed high accuracy, repeatability, and molecular coverage of the method, with over 400 metabolites and lipids detected and identified, including saccharides, amino acids, fatty acids, and glycerophospholipids. Of these, 144 were further confirmed and quantified with LC-MS/MS and standard compounds. We also applied the method to establish lipidomic differences across the isogenic MCF10A cells harboring either WT or MUT *PIK3CA*. Finally, we conducted time-series experiments on hypoxic cells, which revealed significant dynamic changes in metabolism, including lactate accumulation due to anaerobic glycolysis.

## Graphical abstract

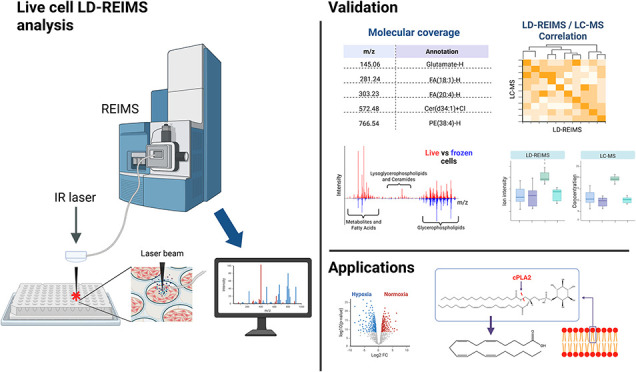

## Introduction

Cell lines are widely used as in vitro model systems across various fields in biochemical and biomedical research, including cancer research, drug screening, and cytotoxicity assessment. They are widely preferred because they are cost-effective, high-throughput, more reproducible and easier to use in comparison to more complex models, such as spheroids, organoids, or animal models, while they provide a virtually unlimited source of biological material.^1,2^ Particularly in cancer research, human cancer-derived cell lines represent the most commonly employed models for investigating the underlying biology and testing hypotheses aimed at improving cancer diagnosis and treatment.^3^

The development of “omics” technologies has facilitated the extensive molecular characterization of cell lines. More recently, metabolomics and lipidomics – which encompass the detection, identification, and quantification of low-molecular-weight molecules and lipids found in a biological system – have been used, alongside genomics, transcriptomics, and proteomics, to establish the molecular phenotype of cells and provide a holistic view of their cellular functions.^4,5^

Metabolites are involved in fundamental biochemical processes, vital for cell survival, including energy production, protein synthesis, redox homeostasis, and signaling.^6^ Various studies have successfully demonstrated the potential of cell culture metabolomics in cancer research, for the identification of effective biomarkers in early detection and prognosis,^7^ studying cancer metabolic reprogramming,^8^ and exposing new therapeutic targets.^9^ At the same time, lipids are also crucial components of the cellular processes, acting as precursors to biologically active molecules. For example, arachidonic acid is a precursor to eicosanoids, which are key regulators of inflammation,^10^ while diacylglycerols (DG) and inositol-1,4,5-trisphosphate (Ins­(1,4,5)­P3) play crucial roles in cellular regulation. DG activates protein kinase C (PKC), while Ins­(1,4,5)­P3 triggers calcium release, both of which initiate a wide range of cellular responses.^11,12^ Therefore, detecting and quantifying lipids and metabolites is essential for understanding cellular mechanisms and establishing a comprehensive readout of the physiological state of a cell.

Mass spectrometry (MS) is the preferred analytical technology used for the metabolomic and lipidomic profiling of cell cultures, especially when hyphenated with chromatographic separation techniques, such as liquid (LC-MS) and gas chromatography (GC-MS).^13–16^ LC-MS in particular is the most widely used technique in metabolomic and lipidomic analyses, due to the extensive metabolite and lipid coverage, structural elucidation capabilities, reproducibility, and sensitivity it provides.^17–19^ While LC-MS can be used for the in-depth biochemical characterization of cells, the required sample preparation is time-consuming and laborious, often involving potent organic solvents for lipid and metabolite extraction that could create a bias in the chemical profiles obtained.^20–24^ Furthermore, the process of detaching cells from the flask or well plate – whether by trypsinization or cell scraping – can lead to metabolite leakage, potentially compromising the robustness and reliability of the results.^20^

Another major challenge in acquiring and interpreting metabolomics and lipidomics data from cell cultures arises from the fact that the molecules detected can reflect products from various stages of metabolic reactions and cellular processes.^25^ This challenge stems from the dynamic nature of cellular metabolism, where metabolite and lipid levels fluctuate rapidly. Real-time biochemical profiling has the potential to address this issue by capturing metabolic changes as they occur, providing a more accurate snapshot of cell metabolism.

Ambient ionization mass spectrometry has emerged in recent years in an effort to address limitations of traditional analytical platforms, due to its ability to ionize analytes from the surface of various types of samples in their native state.^26,27^ Laser Desorption-Rapid Evaporative Ionization Mass Spectrometry (LD-REIMS) is an ambient ionization technique that relies on the desorption of molecules from the surface of biological samples, with minimal or no pretreatment. The technique uses infrared (IR) lasers for the rapid heating and evaporation of biological samples to generate a molecule-rich aerosol, which is subsequently aspirated in the mass spectrometer for analysis^28–30^ (Figure 1). The resulting mass spectra feature predominantly small metabolites and lipid species, such as amino acids, saccharides, fatty acids, glycerophospholipids, lipid dimers, and triacylglycerides.^31^

**Figure 1:**
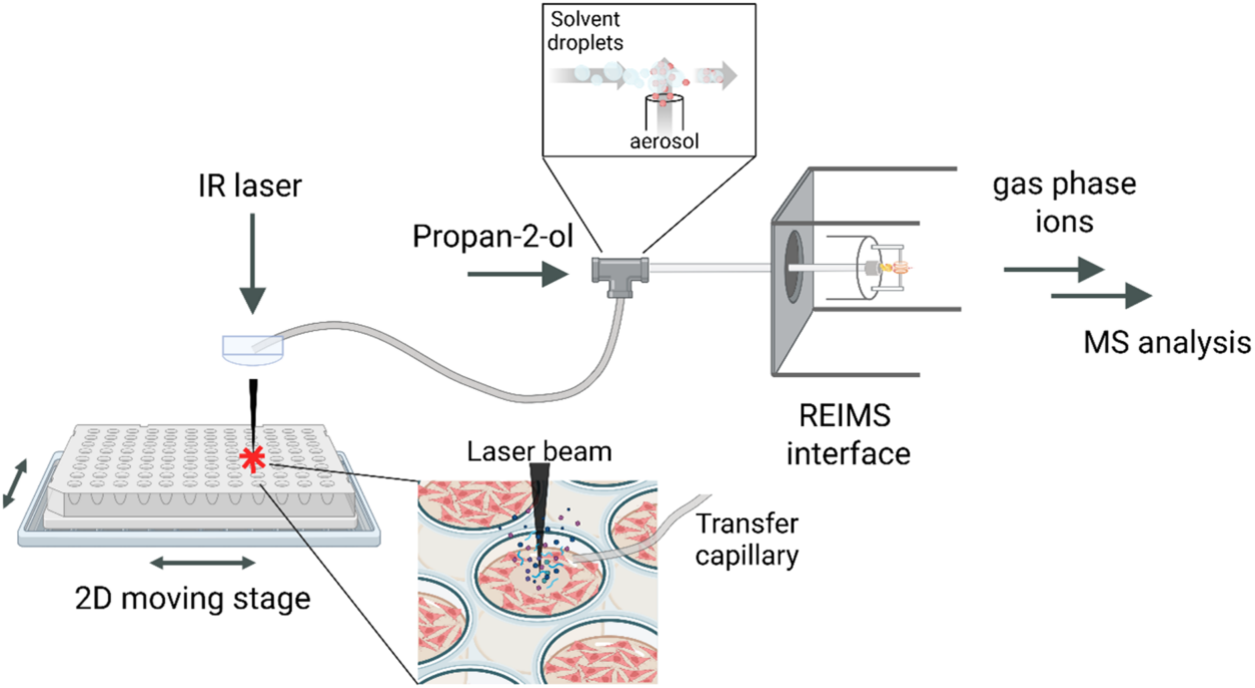
Schematic of the LD-REIMS autosampler. An IR laser is used to desorb cellular material, generating a molecule-rich aerosol. The aerosol is aspirated through a transfer line by the vacuum of the mass spectrometer’s source inlet. Before entering the REIMS interface, the aerosol is introduced orthogonally and dissolved in propan-2-ol to enhance declustering and to solubilize the analytes of interest. The mixture is then passed through the REIMS atmospheric interface, where it collides with a heated surface, causing solvent evaporation and the release of gas-phase ions, which are subsequently analyzed by the mass spectrometer.

Here, we aim to demonstrate the applicability of LD-REIMS incorporated into an automated platform for the high-throughput, real-time biochemical analysis of live and frozen cell monolayers with minimal pretreatment, directly from the cell culture well plates. We performed validation experiments of the method, using a panel of 5 breast cancer (BC) and 5 colorectal cancer (CRC) cell lines, to establish molecular coverage, accuracy, and repeatability. We also compared the LD-REIMS data to the LC-MS profiling and quantification data of the same cell lines. The method was also tested on a previously established metabolic pathway, linking the oncogenic *PIK3CA* mutation to enhanced arachidonic acid metabolism and increased cPLA2 activity.^32^ Finally, we used the epithelial MDCK II renal cell line, cultured under normal and hypoxic conditions and analyzed after 24, 48, 72, and 96 h of incubation, to demonstrate the ability of LD-REIMS to perform timeseries experiments and investigate dynamic changes in the cellular lipidome and metabolome caused by hypoxia.

## Experimental Section

### Cell Culture

Human Breast and Colorectal Cancer Cell lines: Human breast cancer cell lines (BT549, MCF7, MDAMB231, MDAMB468, T47D) and human colorectal cancer cell lines (C2BBE1, LS180, LS513, RKO) were cultured in Dulbecco’s Modified Eagle’s Medium (DMEM) media (Gibco). Human colorectal cancer cell line CL40 was cultured in DMEM/F-12 media (Gibco). Media for all cells was supplemented with 10% Foetal Bovine Serum (FBS) (Gibco). All cells were cultured at 37 °C in a 5% CO_2_ incubator.

*PIK3CA* Mutant and Wild Type MCF10A Breast Cancer Cell lines: *PIK3CA* wild type (WT) and *PIK3CA* mutant (H1047R) isogenic MCF10A cell lines were cultured in DMEM/F-12 growth medium (Gibco) supplemented with 5% horse serum (Gibco), 20 ng/mL epidermal growth factor (EGF) (PeproTech EC, Ltd.), 100 ng/mL cholera toxin, 0.5 mg/mL hydrocortisone and 10 mg/mL insulin (all purchased by Sigma-Aldrich). For experimental conditions, where media free of fatty acid exogenous source was required, horse serum was replaced with 1% fatty acid-free bovine serum albumin (BSA) (Sigma-Aldrich). For cPLA2 inhibition, ASB14780 was used at a concentration of 100 nM and DMSO was used as control treatment. All cell lines were maintained at 37 °C and 5% CO_2_.

MDCK II cell lines cultured under hypoxic and normal conditions: MDCK II cells were purchased from ATCC. Cells were cultured in DMEM growth medium (Gibco) supplemented with 10% FBS, 2 mM l-glutamine, 100 units/mL penicillin, and 100 μg/mL streptomycin (Life Technologies). The cells were incubated under normal and hypoxic conditions at 37 °C, 5% CO_2_, and 20% oxygen and 1% oxygen respectively, for 4 different time points, at 24, 48, 72, and 96 h.

### LD-REIMS Sample Preparation

For the LD-REIMS analysis, the cells were seeded in 96-well plates (Greiner Bio-One) at cell densities ranging from 6,000 to 15,000 cells/well depending on the cell line and incubation time. Multiple wells were seeded for each cell line for repeat measurements. Prior to the analysis, the growth medium was removed, and the cells were washed twice with 150 mM ammonium acetate. MDCK II were washed with 50 μL of HPLC grade water prior to the analysis. Multiple plates with the human breast (BT549, MCF7, MDAMB231, MDAMB468, and T47D) and colorectal cancer cell lines (C2BBE1, CL40, LS180, LS513 and RKO) were cultured. One of them was flash frozen in liquid nitrogen and kept in −80 °C until the day of the analysis and the others were analyzed with LD-REIMS straight after the washes with the ammonium acetate to acquire the mass spectrometric profiles of the live cell monolayers. The frozen cell monolayers were removed from the −80 °C on the day of the analysis and left to thaw in room temperature for 5 min prior to the LD-REIMS analysis.

Cell viability after the ammonium acetate washes was evaluated using trypan blue exclusion. RKO cells were plated at 5,000 cells per well and cultured for 48 h. Following incubation, one plate was washed twice with 150 mM ammonium acetate (pH 7.0) and another with PBS (pH 7.4) under identical conditions. Excess wash solution was removed, and the plates were left at room temperature for approximately 15 min to replicate the duration of the LD-REIMS analysis. Cells were then detached using 0.25% trypsin (Gibco) and mixed 1:1 with trypan blue solution prior to automated counting using a Bio-Rad TC20 automated cell counter. Six wells per condition were measured in duplicate, yielding mean viability ratios of 97.17% for ammonium acetate and 97.25% for PBS, confirming that the washing and incubation steps did not compromise membrane integrity or cell viability prior to LD-REIMS analysis.

### LD-REIMS Profiling

For the LD-REIMS analysis an infrared laser (IR), specifically, an optical parametric oscillator (OPO) (Opolette 2731/3034, OPOTEK – beam width: 4 mm, pulse duration: 7 ns), incorporated as part of an automated prototype LD-REIMS autosampler was used to generate the aerosol from the cell monolayers. The 96-well plates were positioned on a two-dimensional (2D) motorized stage (Thorlabs Inc.), and both the stage movement and laser parameters were controlled through custom LabVIEW-based software, which enabled automated targeting of the selected wells. The laser operated at 2940 nm wavelength, 20 Hz repetition rate, and 5 mW power, corresponding to an irradiance of approximately 160 W cm^–2^ and fluence of 8 J cm^–2^. Each well was analyzed for approximately 10–12 s, as multiple points were sampled within each well to account for spatial heterogeneity and ensure representative spectral acquisition, while there was an approximately 5 s delay between the wells, corresponding to an overall plate analysis time of approximately 25–27 min for a full 96-well plate. In this study, six replicate wells per cell line (60 wells total) were analyzed in randomized order to minimize potential time-dependent variation in signal intensity or molecular profile. Each well received approximately 200–240 laser shots. The laser ablation spot had an approximate diameter of 63 μm, corresponding to a sampled area of 3.1 × 10^3^ μm^2^ (Supporting Information, Figure S2). Considering the average diameters of the breast and colorectal cancer cell lines analyzed (BT549, T47D, MCF7, MDAMB231, MDAMB468, C2BBE1, LS180, LS513, RKO, and CL40), which range between 10–20 μm, this area corresponds to approximately 10–20 cells ablated per ablation point, depending on cell size and local confluence. PEEK tubing was used to transfer the generated aerosol to the mass spectrometer. A Xevo G2-XS QToF mass spectrometer (Waters Corp.), equipped with a REIMS atmospheric interface (MediMass) was used for the mass spectra acquisition. An ACQUITY binary solvent manager (Waters Corp.) was used to introduce propan-2-ol at a flow rate of 0.1 mL/min in the instrument. REIMS data were acquired in negative ion mode (50–1500 Da and 50–1000 Da mass range).

### LD-REIMS Data Processing and Statistical Analysis

The MS data acquisition was performed using MassLynx V4.1 (Waters Corp.). An in-house developed pipeline (Python, Matlab, and R-based) was used for the preprocessing of the mass spectrometric data. The main preprocessing steps included in the pipeline were denoising (using Fast Fourier Transform – FFT), spectral smoothing (using Savitzky–Golay filtering^33^), peak detection, peak alignment, and recalibration, using functions available within the R package MALDIquant. For the recalibration, *m*/*z* 572.4815, which corresponds to the ceramide Cer­(d34:1) (detected as [M+Cl]^−^ adduct) and was present in all the samples, was used as internal lock mass value. The resulting lists of detected peaks were last aligned between different classes and analytical measurements and saved in. csv format for further statistical analysis. Total ion count normalization and variance stabilizing transformation^34^ was performed prior to the statistical analysis. For the statistical analysis, unsupervised and supervised multivariate analyses were used. Principal Component Analysis (PCA) and Volcano plots were generated using Jupyter Notebook. Scatter plot (Figure 2b) and heatmaps (3a and 3b) were generated in Matlab. Ion intensity boxplots were generated in GraphPad Prism.

**Figure 2:**
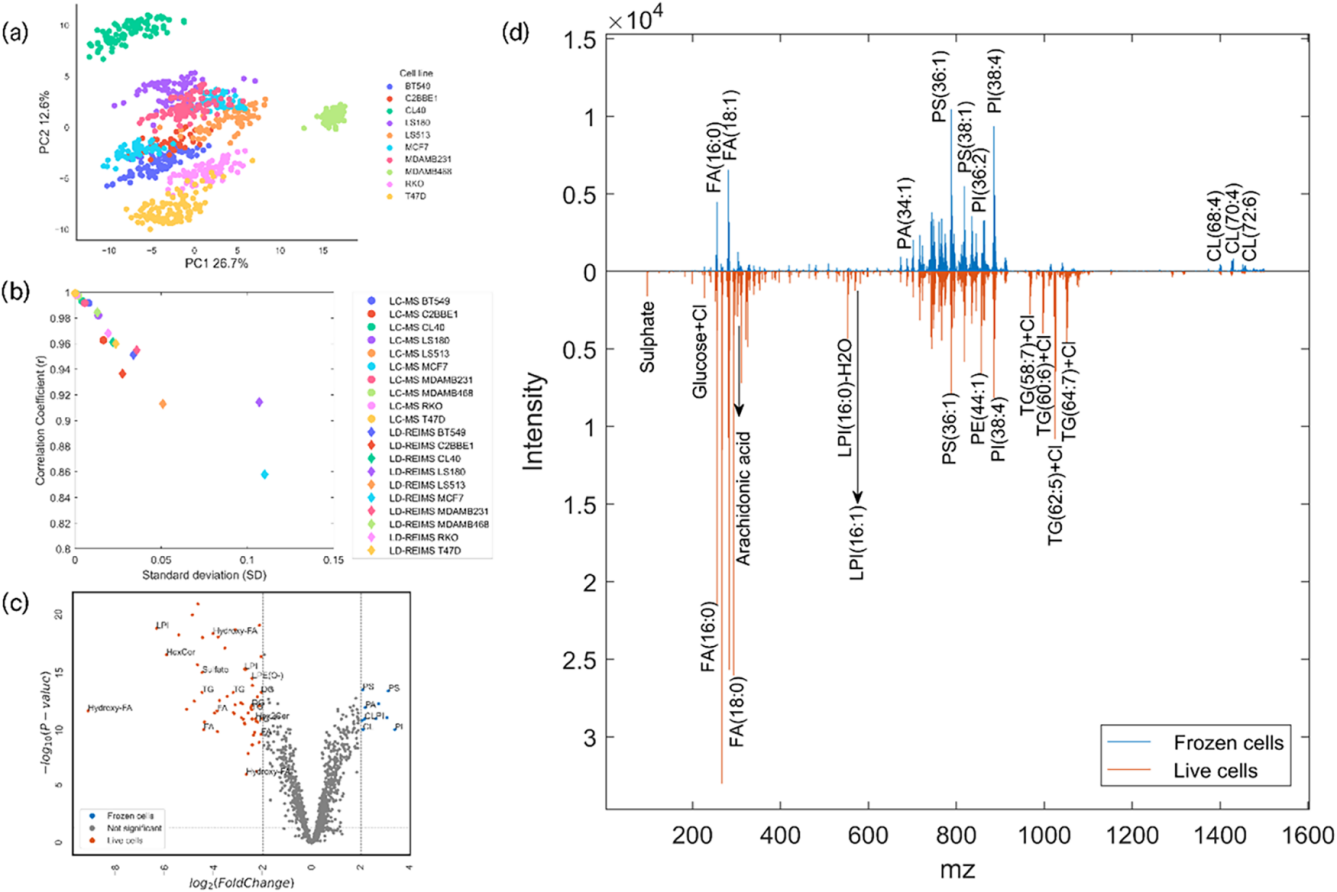
Assessment of repeatability and molecular coverage of LD-REIMS. (a) PCA scores plot of 10 cell lines based on their LD-REIMS spectra, demonstrating good clustering between the replicates of each cell line, indicating high spectral similarity. (b) Scatter plot showing the standard deviation and Pearson correlation coefficient between the biological replicates of each cell line measured with LD-REIMS and LC-MS. For both modalities the correlation coefficients were above 0.9 for the majority of the cell lines (with the exception of the breast cancer cell line MCF7 measured with LD-REIMS). The standard deviations between the replicates are below 0.02 for LC-MS and between 0.015 and 0.11 for the LD-REIMS. (c) Volcano plot showing statistically significant differences (*p* value < 0.05, log_2_(FoldChange) > 2) between the LD-REIMS profiles of the MCF7 cells analyzed as live and frozen cell monolayers. (d) Average LD-REIMS spectra on live and frozen cell monolayers of the MCF7 breast cancer cell line. Both conditions exhibit rich fatty acid and glycerophospholipid regions, while in the live cells, more small metabolites and triacylglycerides (as [M+Cl]^−^) were detected. This demonstrates the importance of real-time analysis with minimal sample preparation, in order to maintain the biochemical integrity of the samples and acquire a representative and accurate snapshot of the underlying cellular metabolism.

### LC-MS Sample Preparation

The human breast and colorectal cancer cell lines were also prepared for (semi)­quantification LC-MS lipidomics and LC-MS/MS amino acid analysis to validate the LD-REIMS results. Five biological replicates were prepared for each cell line and cultured in T75 flasks under the conditions described above. When the cells reached 70–80% confluency they were washed twice with 150 mM ammonium acetate and scraped with 1 mL ammonium acetate to recover the cell extract. The cell extract was split in two microcentrifuge tubes (Eppendorf UK Ltd.), one for the lipid and the other for the amino acid extraction, and underwent centrifugation at 10,000 rpm, at 4 °C, for 5 min to recover the cell pellet. The microcentrifuge tubes were weighted empty and with the cell pellet to establish the cell biomass.

For the lipid extraction, a mixture of 5:1 (v/v) propan-2-ol/water was used (Optima LC/MS grade, Fisher Chemical, Fisher Scientific). The water was added first to lyse the cells and the cell lysate was vortexed for approximately 1 min before the addition of propan-2-ol. The volume was adjusted based on each pellet’s biomass, with 100 μL of solvent mixture added for each 4 mg of cells. The cell lysate was vortexed again and then placed at 4 °C for 2 h to enable protein precipitation. Subsequently, the samples underwent centrifugation at 10,000 rpm, 4 °C, for 10 min, and the supernatant was transferred in HPLC vials for analysis. A pooled quality control (QC) sample was created by combining 5 μL of each sample extract.

For the amino acid extraction, a mixture of 4:1 (v/v) methanol/water was used (Optima LC/MS grade, Fisher Chemical, Fisher Scientific). The water was added first to lyse the cell and the cell lysate was vortexed for approximately 1 min before the addition of methanol. The volume was adjusted based on each pellet’s biomass, with 100 μL of solvent mixture added for each 4 mg of cells. The cell lysates were vortexed again and then sonicated in ice bath for 10 min. Subsequently, the samples underwent centrifugation at 10,000 rpm, 4 °C, for 10 min. A pooled quality control sample (QC) was created by combining 5 μL of each sample extract. 60 μL of the supernatant was transferred to a new microcentrifuge tube (Eppendorf UK Ltd.) containing 5 μL of the stable isotope labeled internal standard mix and left in −20 °C for 20 min. Derivatization using the AccQTag Ultra reagent kit (Waters Corp.) was performed in the cell extracts. For derivatization 10 μL of sample extracts were added in 70 μL borate buffer (pH 8.6) and vortexed, followed by addition of 20 μL of AccQTag Ultra reagent solution. The samples were vortexed again and then placed on a heating block at 55 °C for 10 min. Dilution of 1 μL of sample in 990 μL LC-MS grade water prior to the LC-MS/MS analysis.

### LC-MS Lipidomic Profiling and Semiquantification

For the LC-MS lipidomic profiling and semiquantification, we used the SPLASH II LIPIDOMIX Mass Spec Standard mix (Avanti Polar Lipids), which contains isotope labeled lipid standards of the following lipid classes: phosphatidylcholines (PC), phosphatidylethanolamines (PE), phosphatidylserines (PS), phosphatidylinositols (PI), Lysophosphatidylcholines (LPC), Lysophosphatidylethanolamines (LPE), cholesterol esters, plasmalogens PC and PE lipids, diacylglycerides (DG), triacylglycerides (TG), and sphingomyelins (SM). We supplemented the lipid mixture with Cer­(d18:1/15:0) (d7), palmitic acid (d31), oleic acid (d17) and stearic acid (d35), which represent lipid classes that were not present in the SPLASH II LIPIDOMIX Mass Spec Standard mix. Different concentrations of the lipid standards, diluted in 5:1 (v/v) propan-2-ol/water (Optima LC/MS grade, Fisher Chemical, Fisher Scientific) to create calibration curves.

LC-MS analysis was performed using an ACQUITY UPLC system (Waters Corp.) coupled to a Xevo G2-S QToF mass analyzer (Waters Corp.) using a LC-MS lipidomics assay developed and validated by the National Phenome Centre (NPC, Imperial College London).^35^ The column used for the chromatographic separation was a BEH C8 column, 1.7 μm, 2.1 × 100 mm (Waters Corp.) which was kept at 55 °C during the analysis. Mobile phase A consisted of water/propan-2-ol/acetonitrile in a ratio of 2:1:1 (v/v/v), with a buffer system of 5 mM ammonium acetate, 0.05% acetic acid and 20 μM phosphoric acid (Sigma-Aldrich). Mobile B consisted of propan-2-ol/acetonitrile in ratio of 1:1 (v/v), 5 mM ammonium acetate (Sigma-Aldrich) and 0.5% acetic acid (Sigma-Aldrich). The MS spectra were acquired at 50–2000 Da mass range and at 0.1 scan/sec in continuous mode, in negative ion mode.

Data acquired using MassLynx V4.1 (Waters Corp.) and spectral processing and ion peak area calculation was conducted using MZmine2.^36^

### LC-MS/MS Amino Acid Quantification Assay

LC-MS/MS analysis and quantification of amino acids was performed by the National Phenome Centre (Imperial College) using an ACQUITY UPLC system coupled with a TQ-S (Waters Corp.). The experimental procedure was performed based on the validated method as described previously by Gray et al.^37^ Standard compounds purchased from Sigma-Aldrich were used for quantification.

Data acquisition was conducted using MassLynx V4.1 (Waters Corp.) and data processing and sample concentration calculations was performed with TargetLynx Application Manager (Waters Corp.).

### Metabolite and Lipid Identification

Lipid and metabolite annotation was performed using in-house and online databases (including LIPIDMAPS, METLIN and HMDB) based on the accurate mass, elemental composition, and isotopic ratio analysis. For certain lipids and amino acids, identification was performed by LC-MS/MS analysis, based on the retention time and the manual MS/MS spectra interpretation.

### LD-REIMS and LC-MS Data Correlation

Common lipids and metabolites found in LD-REIMS and LC-MS were compared across the 5 colorectal and 5 breast cancer cell lines. Canonical correlation analysis was performed, specifically the cosine similarity was calculated between the LD-REIMS normalized ion intensities and the LC-MS ion concentrations for the same species to establish the correlation between modalities.

## Results and Discussion

### Repeatability and Molecular Coverage

To assess the repeatability of the LD-REIMS measurements we used a panel of 10 cell lines, 5 breast (BT549, MCF7, MDAMB231, MDAMB468, T47D) and 5 colorectal (C2BBE1, CL40, LS180, LS513, RKO) cancer cell lines, measured by LD-REIMS as live cell monolayers, directly after the removal of the culture well plates from the incubator. Principal Component Analysis (PCA) demonstrated clustering between the replicates of the different cell lines, which indicates reproducibility of their LD-REIMS profiles (Figure 2a). Pearsons correlation coefficients and standard deviations were also calculated between the LD-REIMS spectra of each cell line and compared to LC-MS. As shown in Figure 2b for both LD-REIMS and LC-MS, correlation coefficients were above 0.9 for the majority of the cell lines – with the exception of MCF7, which was 0.86 in LD-REIMS – while the standard deviations for each cell line were below 0.02 for LC-MS and between 0.015 and 0.11 for LD-REIMS. Replicate wells analyzed at different time points throughout the run showed no time-dependent spectral differences, as evidenced by the consistently high Pearson correlation coefficients and low variation across replicates, confirming the stability of the LD-REIMS measurements. While LC-MS exhibited better correlation coefficients and standard deviations between the spectra of each cell line, LD-REIMS displayed comparable values, indicating good repeatability and spectral/chemical similarity between replicates.

Mass spectra from the same panel of cell lines were also used to establish the molecular coverage of LD-REIMS. 2,526 peaks were detected in the live cell monolayers – after the data preprocessing and peak picking – from which 402 metabolites and lipids (excluding isotopic peaks) were tentatively identified (Supporting Information, Table S1), based on accurate mass, isotopic pattern, biological relevance, and ppm error from the theoretical *m*/*z* value (identifications with >15 ppm error were excluded).

Notably, the LD-REIMS spectra featured a significant number of chlorinated adducts ([M+Cl]^−^) of lipids and metabolites that we would not usually be able to detect as negative ions, such as glycerophosphatidylcholines (PC), sphingomyelins (SM), and triacylglycerides (TG). From the 402 metabolites and lipids annotated from the LD-REIMS spectra of the live cell monolayers, 211 were detected as deprotonated ([M-H]^−^) ions, 160 as chlorinated adducts ([M+Cl]^−^), and 31 as deprotonated ions with loss of a water molecule ([M-H_2_O-H]^−^).

Significant differences were observed between the spectra of the live and frozen cell monolayers, with the live cells exhibiting much richer small metabolite and triacylglyceride (TG) content (Figure 2d). Even though flash freezing generally helps to maintain the chemical integrity of biological samples, and has been widely used in biological research for cryopreservation of tissues and cells, it has been found that freeze–thaw cycles can cause physical and chemical alterations in the cells and disintegration of their plasma membrane.^38^ Consistent with this, optical microscopy (Supporting Information, Figure S1) showed that frozen monolayers display pronounced freezing artifacts, cellular debris, and rupture of the plasma membrane, whereas live cell monolayers washed with ammonium acetate or PBS maintained intact morphology and a homogeneous cell population. These structural differences explain the distinct spectral profiles, with frozen cell spectra reflecting the release and mixing of intracellular and extracellular components from disrupted membranes, while live cell spectra represent the compartmentalized molecular composition of intact cells.

### LD-REIMS and LC-MS Correlation

We performed quantitative LC-MS lipidomic and amino acid analyses, on the same 5 breast and 5 colorectal cancer cell lines we analyzed with LD-REIMS, to confirm metabolite and lipid identifications, but also to establish if the LD-REIMS ion intensities correlate with the molecules’ abundances/concentrations.

Overall, we assessed 144 amino acids and lipids that were found to be common between the two modalities (Tables S2 and S3). Figure 3a,b demonstrates correlations between the molecular classes measured. Specifically, Figure 3a shows the correlation – based on Cosine similarity – between the LD-REIMS ion intensities (*x* axis) and the LC-MS established concentrations (*y* axis) averaged by molecular class. The majority of the classes demonstrated high correlation (>0.8) between the LD-REIMS ion intensities and the LC-MS concentrations. Figure 3c–e features three example molecules – histidine, Cer­(d34:1), and PS(38:3) – from the 144 used for this comparison, where the top boxplots show the normalized LD-REIMS ion intensities across the 10 cell lines and bottom boxplots show the respective LC-MS-established concentrations. Similar distribution patterns between LD-REIMS and LC-MS were observed across all three examples. Histidine was found to be more abundant in the CL40 and RKO cell lines (Figure 3c) and Cer­(d34:1) in CL40 and LS513, while PS(38:3) was more enriched in LS513 and MDAMB468, but less abundant in RKO and T47D cell lines.

**Figure 3:**
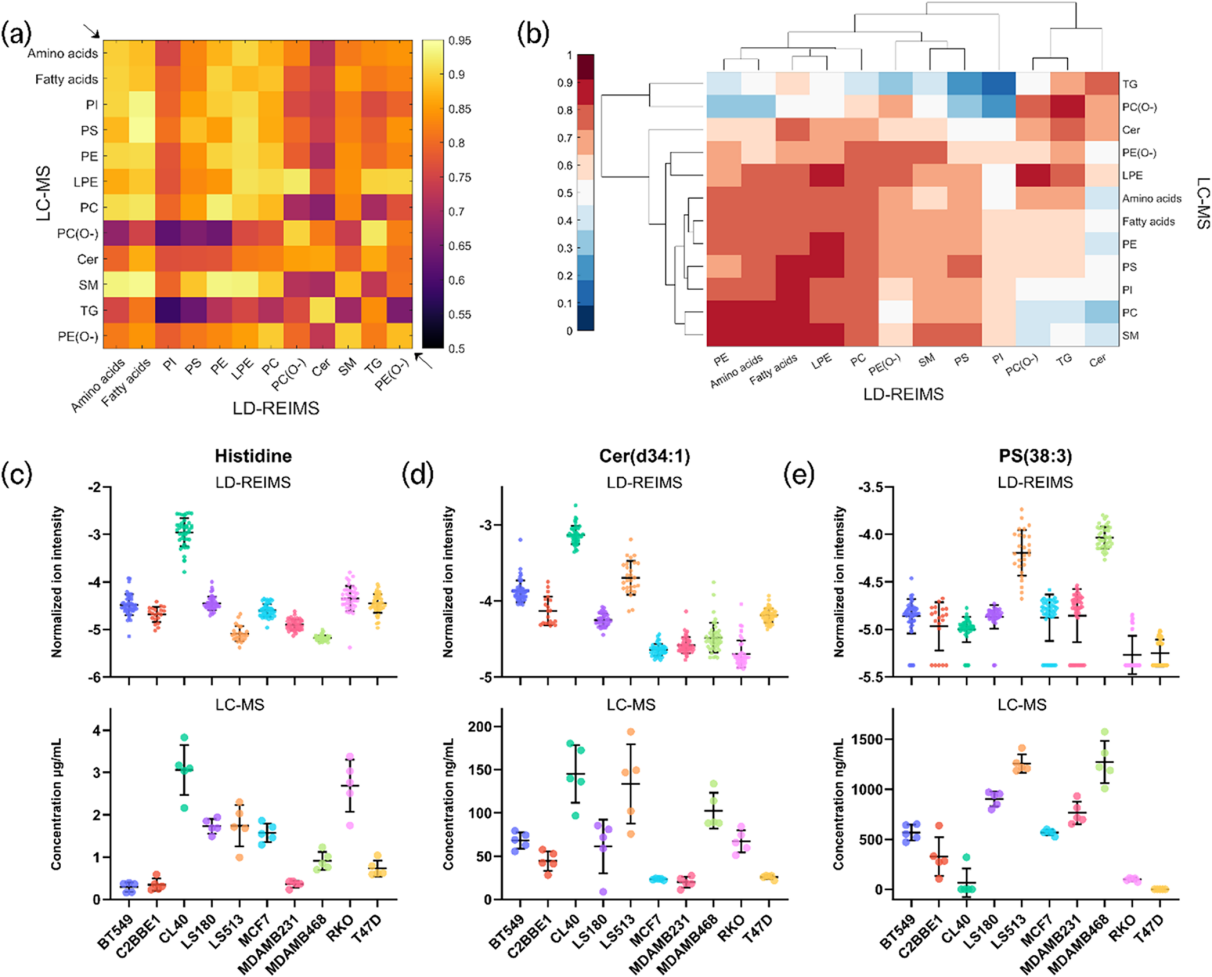
Correlation of the LD-REIMS ion intensities with the LC-MS-established concentrations of amino acids and lipids found across 5 breast and 5 colorectal cancer cell lines. (a) Correlation heatmap based on Cosine similarity between LD-REIMS relative ion intensities and LC-MS concentrations of the molecular classes detected in both modalities. The majority of the molecular classes demonstrate cosine similarity >0.8 (as it can be observed in the diagonal line between the *x* and *y* axis of the heatmap, indicated with the arrows). (b) Heatmap illustrating the hierarchical clustering of molecular classes in LD-REIMS (*x* axis) and LC-MS (*y* axis) using Manhattan distance. Both modalities seem to identify a large cluster with correlations of 0.7 and above for the PE, PC, amino acids, and fatty acids, while a smaller cluster is also present on both modalities between TG and PC­(O−). (c–e) Boxplots of histidine, Cer­(d34:1), and PS(38:4) showing the LD-REIMS normalized ion intensities (top plots) versus the LC-MS concentrations (bottom plots) across the 10 cell lines.

The consistent relationship between the two methods suggests that LD-REIMS ion ratios provide a reliable indication of the relative abundances of metabolites and lipids across different cell groups, establishing LD-REIMS as an analytical approach that combines high molecular coverage and throughput with semiquantitative performance. The reproducible relationship between ion intensity and analyte abundance supports its use for comparative metabolomic and lipidomic analyses, bridging qualitative ambient MS profiling and quantitative LC-MS workflows.

Figure 3b shows a heatmap of hierarchical clustering of molecular classes in LD-REIMS (*x* axis) and LC-MS (*y* axis) using Manhattan distance. In both modalities, we observed two main clusters, with TG and PC­(O−) lipids clustering together – along with Cer in LD-REIMS −, and a larger cluster between amino acids, fatty acids, PE, and PC. Plasmalogens – primarily plasmenylethanolamine – PE­(P-) – and plasmenylcholine – PC­(P-) – are the most common subclass of ether lipids – PE­(O−) and PC­(O−) respectively – and they are characterized by a vinyl ether bond in the *sn1* position of the glycerol moiety.^39^ Deficiency of plasmalogens has been found to significantly affect and decrease the formation of lipid droplets, which primarily consist of TGs,^40^ which could justify the correlation between PC­(P-)/PC­(O−) and TG in both LD-REIMS and LC-MS. The clustering of PEs and PCs – both major components of the cellular membranes – is expected as the two lipid classes are directly connected via two main pathways for the biosynthesis of PCs from PEs, either via PE methylation,^41^ or via the Kennedy pathway if exogenous choline is available.^42,43^ Similarly, fatty acids are key components in the PE and PC metabolism, since they are building blocks of the glycerophospholipids, incorporated as their fatty acyl chains. Amino acids are also involved in PE and PC metabolism, with a characteristic example being the exchange of serine with choline or ethanolamine in PC and PE respectively for the biosynthesis of PS.^44^ Methionine, is also directly involved in PC biosynthesis as it is the precursor of S-adenosylmethionine (SAM) which facilitates the PE methylation.^43^ The fact that both modalities – LD-REIMS and LC-MS – feature similar clusters between molecular classes, that are structurally and/or functionally related, demonstrates the accuracy of the results acquired by LD-REIMS.

### Time-Series Experiments

To demonstrate the method’s ability to perform time-series experiments and highlight the benefit of live cell analysis with mass spectrometry, we used MDCK II cells cultured under hypoxic and normoxic conditions and measured their metabolic profiles with LD-REIMS at 24, 48, 72, and 96 h of incubation. In the PCA scores plot (Figure 4a), a time-dependent pattern is observed between the two conditions, with the variance gradually increasing between normoxic and hypoxic cells from 48 to 96 h. At 24 h, no difference between the two conditions was observed. One of the metabolites significantly increased in hypoxic cells was lactic acid, detected as [M-H]^−^. Lactic acid intensities remained similar at 24 and 48 h, but differences were noted at 72 h and became more pronounced at 96 h, where lactate was increased by more than 2-fold in hypoxic cells (Figure 4b). These observations indicate that anaerobic glycolysis occurs in the hypoxic cells, resulting in pyruvate getting converted into lactate, instead of being further oxidized through entering the citric acid cycle. The volcano plots for each time point (Figure 4c) show the lipids and metabolites found to be significantly different – *p* < 0.05 and log_2_(fold change) > 2 – between the two conditions. Glucose, primarily detected as [M+Cl]^−^, was also increased in hypoxic cells at 48 and 72 h, with no significant difference observed at 96 h. This finding aligns with the literature, indicating that cells in hypoxia increase glucose uptake, which ultimately undergoes anaerobic glycolysis, leading to lactate accumulation.^45^

**Figure 4:**
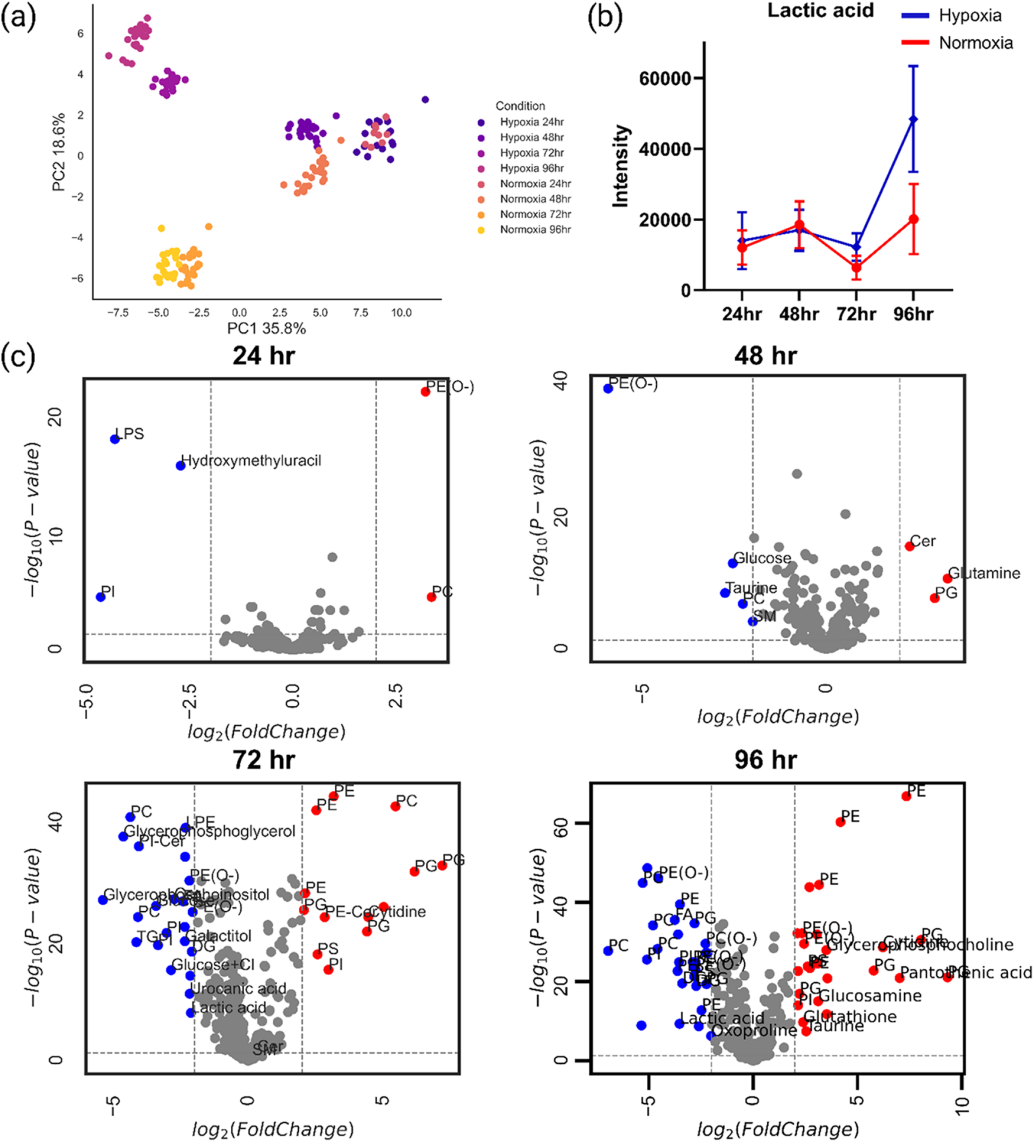
Time-series experiments and metabolic differences between hypoxic and normoxic cells (a) PCA scores plot of the MDCK II hypoxic and normoxic cells at the 4 time points. At 24 h the hypoxic and normoxic cells seems to cluster together indicating high spectral similarity. With increasing incubation times, however, they exhibit clear separation, which maximizes at 96 h. (b) One of the main metabolites responsible for the time-dependent pattern observed in the PCA scores plot is lactic acid. Lactic acid’s abundance is similar in both conditions at 24 and 48 h, however, at 72 h it starts increasing in the hypoxic cells and at 96 h it is significantly increased under hypoxia. This is consistent with hypoxic cells accumulating lactic acid due to increased anaerobic glycolysis. (c) Volcano plots of normoxic and hypoxic cells at 24, 48, 72, and 96 h. Metabolic and lipidomic differences between the two conditions significantly increase at 72 and 96 h in comparison to 24 and 48 h, with lactic acid, glucose, and ether glycerophospholipids (PE­(O−) and PC­(O−)) – among others – significantly increased in the hypoxic cells.

### Oncogenic *PIK3CA* Promotes Enhanced Arachidonic Acid Metabolism

The method was tested in a previously established metabolic pathway, linking oncogenic *PIK3CA* to enhanced arachidonic acid metabolism and increased cPLA2 activity.^32^ Isogenic *PIK3CA* mutant and wild type (WT) breast epithelial MCF10A cell lines were analyzed with LD-REIMS as live cell monolayers. As shown in Figure 5b, arachidonic and linoleic acids were found to increase in the mutant cells when compared to the wild type, while glycerophospholipids containing arachidonate, such as PI(18:0_20:4) were found decreased in the mutant cells, indicating enhanced activity of the cPLA2 enzyme in the oncogenic *PIK3CA* mutant cells. Cells, treated with the cPLA2 inhibitor ASB14780 were also analyzed and the arachidonic acid levels were found to be significantly lower in the mutant cells, as opposed to the wild type cells, where it remained unchanged, indicating again the link between oncogenic *PIK3CA* with the cPLA2 activity and the arachidonic acid metabolism.

**Figure 5:**
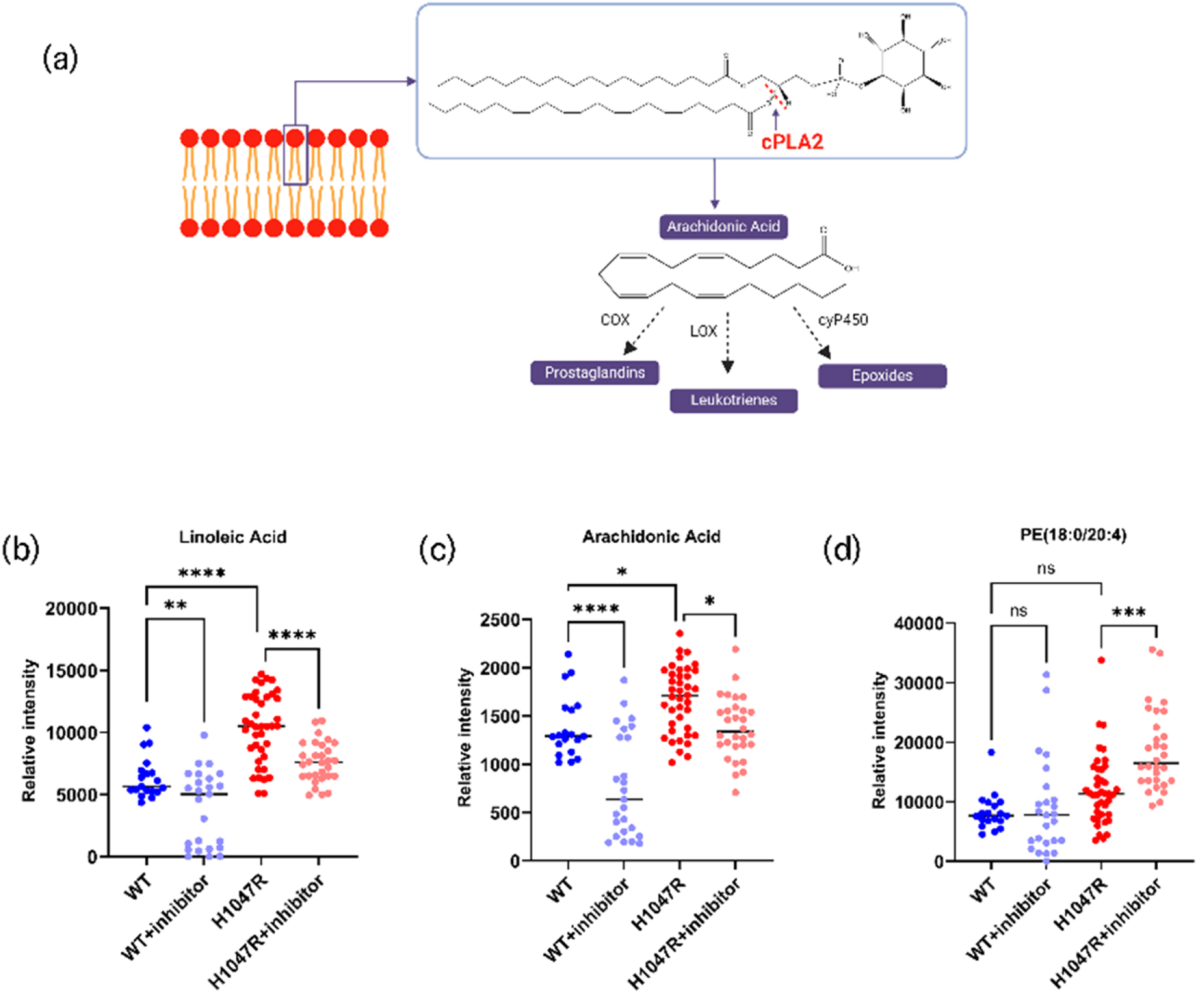
Oncogenic *PIK3CA* mutation drives arachidonic acid increase in breast and colorectal cancer cell lines. (a) The cPLA2 enzyme catalyzes the cleavage of the fatty acyl chains at the sn2 position of membrane glycerophospholipids. (b–d) Ion intensity boxplots of the polyunsaturatred omega-6 fatty acids, linoleic (FA(18:2)) and arachidonic acid, and PE(18:0_20:4) lipid. The boxplots show the LD-REIMS ion intensity distributions in wild type (WT) and *PIK3CA* mutant (H1047R) breast epithelial MCF10A cells, with and without treatment with a cPLA2 inhibitor. Arachidonic and linoleic acids were significantly increased (*p* values < 0.05 and 0.0001 respectively) in H1047R *PIK3CA* mutant cells compared to their wild type counterparts, which supports the link that was previously shown by Koundouros et al. between oncogenic *PIK3CA* mutation and enhanced arachidonic acid metabolism and cPLA2 activity. Additionally, in the PE­(18:0/20:4) lipid which contains arachidonate, significant increase (*p* value < 0.001) is shown in the H1047R mutant cells after treatment with the cPLA2 inhibitor ASB14780, while in the WT cells the levels of the lipid remain the same and difference is not significant, indicating preferential cleavage of the arachidonate containing lipids in the *PIK3CA* mutant cells.

## Conclusions

In summary, we have developed an ambient ionization method for high-throughput metabolomic and lipidomic profiling of live or frozen cell monolayers directly from the well plates in which they are cultured. The method requires only a small number of cells (approximately 10–20 cells depending on the cell line and confluence), significantly reducing the time needed to culture large cell numbers. Additionally, the absence of extensive sample preparation further increases throughput and minimizes user-dependent variations. We detected a wide range of metabolite and lipid classes, including amino acids, saccharides, fatty acids, and complex lipids, which were further validated using LC-MS/MS and standard compounds. Notably, many of the ions detected were chlorinated adducts, extending the range of molecular classes detectable in negative ion mode to molecules that would not normally be identified as deprotonated ions, such as saccharides, PCs, and TGs. This allows comprehensive biochemical coverage of lipids and metabolites in a single measurement.

Therefore, from an analytical perspective, LD-REIMS provides a robust and reproducible platform for rapid metabolomic and lipidomic profiling of intact cells, offering a high-throughput approach and molecular coverage that extend the capabilities of current ambient ionization techniques. Its consistent ion-intensity patterns across replicate samples highlight the method’s potential for reproducible relative quantification, positioning LD-REIMS as a complementary method between qualitative ambient MS and quantitative LC-MS workflows.

To enable more accurate real-time cell measurements, a sterile box capable of controlling temperature and CO_2_ conditions and an automated liquid handling system could be incorporated into the LD-REIMS autosampler. This would facilitate continuous live-cell monitoring, similar to how live-cell fluorescence and bright-field imaging systems operate, allowing dynamic profiling of metabolites and lipids throughout the cell cycle.

## Supplementary Material



## Notes

Table S1: Table with annotations of the *m*/*z* values detected from the LD-REIMS analysis of the 5 breast and 5 colorectal cancer cell lines that were used for the validation of the method. Figure S1: Brightfield images of live and frozen RKO cell monolayers washed with 150 mM ammonium acetate or PBS, showing intact morphology in live conditions and loss of membrane integrity following freezing. Figure S2: Brightfield images of RKO cell monolayer before and after LD-REIMS laser ablation, showing a single ablation spot (approximately 63 μm diameter) corresponding to the sampling of approximately 10–20 cells. Table S2: Table with the lipids validated and quantified with LC-MS. Table S3: Table with the amino acids validated and quantified with LC-MS/MS and standard compounds (PDF)

## Abbreviations

BSA: bovine serum albumin
cPLA2: cytosolic phospholipase A2
CRC: colorectal cancer
DG: diacylglycerol
DMEM: Dulbecco’s Modified Eagle’s Medium
DMSO: dimethyl sulfoxide
EGF: epidermal growth factor
FBS: fetal bovine serum
FFT: Fast Fourier Transform
GC-MS: gas chromatography–mass spectrometry
Ins­(1,4,5)­P3: inositol-1,4,5-trisphosphate
IR: infrared
LC-MS: liquid chromatography–mass spectrometry
LC-MS/MS: liquid chromatography-tandem mass spectrometry
LD-REIMS: laser desorption-rapid evaporative ionization mass spectrometry
LPC: lysophosphatidylcholine
LPE: lysophosphatidylethanolamine
MDCK: Madin-Darby Canine Kidney
MS: mass spectrometry
MS/MS: tandem mass spectrometry
OPO: optical parametric oscillator
PCA: principal component analysis
PC: phosphatidylcholine
PC­(O−): ether-linked phosphatidylcholine
PC­(P-): plasmenylcholine
PE: phosphatidylethanolamine
PE­(O−): ether-linked phosphatidylethanolamine
PE­(P-): plasmenylethanolamine
PI: phosphatidylinositol
PKC: protein kinase C
PS: phosphatidylserine
QC: quality control
QToF: quadrupole time-of-flight
SAM: S-adenosylmethionine
SM: sphingomyelin
SVM: support vector machine
TG: triacylglyceride
UPLC: ultra performance liquid chromatography
WT: wild type

## References

[1] Mirabelli P., Coppola L., Salvatore M. (2019). Cancer Cell Lines Are Useful Model
Systems for Medical Research. Cancers.

[2] Li, Z. In Vitro Micro-Tissue and -Organ Models for Toxicity Testing 2nd ed.; Moo-Young, M. B. T.-C. B. , Ed.; Academic Press: Burlington, 2011; pp 551–563.

[3] Gillet J.-P., Varma S., Gottesman M. M. (2013). The Clinical
Relevance of Cancer
Cell Lines. J. Natl. Cancer Inst..

[4] Zhang A., Sun H., Xu H., Qiu S., Wang X. (2013). Cell Metabolomics. OMICS.

[5] Jaiswal, A. ; Gautam, P. ; Pietilä, E. A. ; Timonen, S. ; Nordström, N. ; Sipari, N. ; Tanoli, Z. ; Lehti, K. ; Wennerberg, K. ; Aittokallio, T. Multi-Modal Meta-Analysis of Cancer Cell Line Omics Profiles Identifies ECHDC1 as a Novel Breast Tumor Suppressor bioRxiv 2020 10.1101/2020.01.31.929372. PMC7983037 33750001

[6] Zhu J., Thompson C. B. (2019). Metabolic
Regulation of Cell Growth and Proliferation. Nat. Rev. Mol. Cell Biol..

[7] Jain M., Nilsson R., Sharma S., Madhusudhan N., Kitami T., Souza A. L., Kafri R., Kirschner M. W., Clish C. B., Mootha V. K. (2012). Metabolite Profiling
Identifies a
Key Role for Glycine in Rapid Cancer Cell Proliferation. Science.

[8] Piskounova E., Agathocleous M., Murphy M. M., Hu Z., Huddlestun S. E., Zhao Z., Leitch A. M., Johnson T. M., DeBerardinis R. J., Morrison S. J. (2015). Oxidative Stress Inhibits Distant Metastasis by Human
Melanoma Cells. Nature.

[9] Xiao Y., Ma D., Yang Y.-S., Yang F., Ding J.-H., Gong Y., Jiang L., Ge L.-P., Wu S.-Y., Yu Q., Zhang Q., Bertucci F., Sun Q., Hu X., Li D.-Q., Shao Z.-M., Jiang Y.-Z. (2022). Comprehensive Metabolomics
Expands Precision Medicine for Triple-Negative Breast Cancer. Cell Res..

[10] Balazy M. (2004). Eicosanomics:
Targeted Lipidomics of Eicosanoids in Biological Systems. Prostaglandins Other Lipid Mediat..

[11] Nishizuka Y. (1992). Intracellular
Signaling by Hydrolysis of Phospholipids and Activation of Protein
Kinase C. Science.

[12] Hannun Y. A., Obeid L. M. (2008). Principles of Bioactive
Lipid Signalling: Lessons from
Sphingolipids. Nat. Rev. Mol. Cell Biol..

[13] Rustam Y.
H., Reid G. E. (2018). Analytical
Challenges and Recent Advances in Mass Spectrometry
Based Lipidomics. Anal. Chem..

[14] Wishart D. S., Cheng L. L., Copié V., Edison A. S., Eghbalnia H. R., Hoch J. C., Gouveia G. J., Pathmasiri W., Powers R., Schock T. B., Sumner L. W., Uchimiya M. (2022). NMR and Metabolomics—A
Roadmap for the Future. Metabolites.

[15] Dettmer K., Aronov P. A., Hammock B. D. (2007). Mass Spectrometry-Based
Metabolomics. Mass Spectrom. Rev..

[16] Kontiza A., Gerichten J. von., Saunders K. D. G., Spick M., Whetton A. D., Newman C. F., Bailey M. J. (2024). Single-Cell Lipidomics: An Automated
and Accessible Microfluidic Workflow Validated by Capillary Sampling. Anal. Chem..

[17] Gika H., Virgiliou C., Theodoridis G., Plumb R. S., Wilson I. D. (2019). Untargeted
LC/MS-Based Metabolic Phenotyping (Metabonomics/Metabolomics): The
State of the Art. J. Chromatogr. B.

[18] Hu T., Zhang J.-L. (2018). Mass-Spectrometry-Based
Lipidomics. J. Sep. Sci..

[19] Plumb R. S., Gethings L. A., Rainville P. D., Isaac G., Trengove R., King A. M., Wilson I. D. (2023). Advances
in High Throughput LC/MS
Based Metabolomics: A Review. TrAC Trends Anal.
Chem..

[20] Rombouts C., De Spiegeleer M., Van Meulebroek L., De Vos W. H., Vanhaecke L. (2019). Validated
Comprehensive Metabolomics and Lipidomics Analysis of Colon Tissue
and Cell Lines. Anal. Chim. Acta.

[21] Peng W., Tan S., Xu Y., Wang L., Qiu D., Cheng C., Lin Y., Liu C., Li Z., Li Y., Zhao Y., Li Q. (2018). LC-MS/MS Metabolome
Analysis Detects the Changes in the Lipid Metabolic
Profiles of DMMR and PMMR Cells. Oncol. Rep..

[22] Yusufi F. N. K., Lakshmanan M., Ho Y. S., Loo B. L. W., Ariyaratne P., Yang Y., Ng S. K., Tan T. R. M., Yeo H. C., Lim H. L., Ng S. W., Hiu A. P., Chow C. P., Wan C., Chen S., Teo G., Song G., Chin J. X., Ruan X., Sung K. W. K., Hu W. S., Yap M. G. S., Bardor M., Nagarajan N., Lee D. Y. (2017). Mammalian Systems
Biotechnology Reveals Global Cellular Adaptations in a Recombinant
CHO Cell Line. Cell Syst..

[23] Yeo H. C., Chen S., Ho Y. S., Lee D. Y. (2018). An LC–MS-Based
Lipidomics Pre-Processing Framework Underpins Rapid Hypothesis Generation
towards CHO Systems Biotechnology. Metabolomics.

[24] Moosmang S., Pitscheider M., Sturm S., Seger C., Tilg H., Halabalaki M., Stuppner H. (2019). Metabolomic Analysis—Addressing
NMR and LC-MS Related Problems in Human Feces Sample Preparation. Clin. Chim. Acta.

[25] Wang R., Li B., Lam S. M., Shui G. (2020). Integration
of Lipidomics and Metabolomics
for In-Depth Understanding of Cellular Mechanism and Disease Progression. J. Genet. Genomics.

[26] Huang M.-Z., Yuan C.-H., Cheng S.-C., Cho Y.-T., Shiea J. (2010). Ambient Ionization
Mass Spectrometry. Annu. Rev. Anal. Chem..

[27] Takáts Z., Wiseman J. M., Gologan B., Cooks R. G. (2004). Mass Spectrometry
Sampling under Ambient Conditions with Desorption Electrospray Ionization. Science.

[28] Simon D., Horkovics-Kováts G. S., Xiang Y., Battle R. A., Wang Y., Abda J., Papanastasiou D., Stavrakaki S. M., Ho H.-Y., Wang H., Schäffer R., Karancsi T., Mroz A., Pap I., Lagache L., Balog J., Fournier I., Murray R. T., Bunch J., Takáts Z. (2025). Subcellular-Resolution Molecular
Pathology by Laser
Ablation–Rapid Evaporative Ionization Mass Spectrometry. Anal. Chem..

[29] Schäfer K., Dénes J., Albrecht K., Szaniszló T., Balogh J., Skoumal R., Katona M., Tóth M., Balogh L., Takáts Z. (2009). In Vivo, in
Situ Tissue Analysis
Using Rapid Evaporative Ionization Mass Spectrometry. Angew. Chem. - Int. Ed..

[30] Jones E. A., Simon D., Karancsi T., Balog J., Pringle S. D., Takats Z. (2019). Matrix Assisted Rapid Evaporative Ionization Mass Spectrometry. Anal. Chem..

[31] Golf O., Strittmatter N., Karancsi T., Pringle S. D., Speller A. V. M., Mroz A., Kinross J. M., Abbassi-Ghadi N., Jones E. A., Takats Z. (2015). Rapid Evaporative
Ionization Mass
Spectrometry Imaging Platform for Direct Mapping from Bulk Tissue
and Bacterial Growth Media. Anal. Chem..

[32] Koundouros N., Karali E., Tripp A., Valle A., Inglese P., Perry N. J. S., Magee D. J., Anjomani Virmouni S., Elder G. A., Tyson A. L., Dória M. L., van Weverwijk A., Soares R. F., Isacke C. M., Nicholson J. K., Glen R. C., Takats Z., Poulogiannis G. (2020). Metabolic
Fingerprinting Links Oncogenic PIK3CA with Enhanced Arachidonic Acid-Derived
Eicosanoids. Cell.

[33] Savitzky A., Golay M. J. E. (1964). Smoothing and
Differentiation of Data by Simplified
Least Squares Procedures. Anal. Chem..

[34] Veselkov K. A., Vingara L. K., Masson P., Robinette S. L., Want E., Li J. V., Barton R. H., Boursier-Neyret C., Walther B., Ebbels T. M., Pelczer I., Holmes E., Lindon J. C., Nicholson J. K. (2011). Optimized Preprocessing of Ultra-Performance
Liquid Chromatography/Mass Spectrometry Urinary Metabolic Profiles
for Improved Information Recovery. Anal. Chem..

[35] Lewis, M. ; Chekmeneva, E. ; Camuzeaux, S. ; Sands, C. ; Yuen, A. ; David, M. ; Salam, A. ; Chappell, K. ; Cooper, B. ; Haggart, G. ; Maslen, L. ; Gomez-Romero, M. ; Horneffer-van der Sluis, V. ; Correia, G. ; Takats, Z. An Open Platform for Large Scale LC-MS-Based Metabolomics ChemRxiv 2022 10.26434/chemrxiv-2022-nq9k0.

[36] Pluskal T., Castillo S., Villar-Briones A., Orešič M. (2010). MZmine 2:
Modular Framework for Processing, Visualizing, and Analyzing Mass
Spectrometry-Based Molecular Profile Data. BMC
Bioinf..

[37] Gray N., Plumb R. S., Wilson I. D., Nicholson J. K. (2019). A Validated
UPLC-MS/MS Assay for the Quantification of Amino Acids and Biogenic
Amines in Rat Urine. J. Chromatogr. B.

[38] Ahn H. J., Sohn I. P., Kwon H. C., Jo D. H., Park Y. D., Min C. K. (2002). Characteristics
of the Cell Membrane Fluidity, Actin
Fibers, and Mitochondrial Dysfunctions of Frozen-Thawed Two-Cell Mouse
Embryos. Mol. Reprod. Dev..

[39] Dean J. M., Lodhi I. J. (2018). Structural and Functional
Roles of Ether Lipids. Protein Cell.

[40] Brites P., Ferreira A. S., da Silva T. F., Sousa V. F., Malheiro A. R., Duran M., Waterham H. R., Baes M., Wanders R. J. A. (2011). Alkyl-Glycerol
Rescues Plasmalogen Levels and Pathology of Ether-Phospholipid Deficient
Mice. PLoS One.

[41] Bremer J., Greenberg D. M. (1961). Methyl Transfering Enzyme System
of Microsomes in the
Biosynthesis of Lecithin (Phosphatidylcholine). Biochim. Biophys. Acta.

[42] Kennedy E. P., Weiss S. B. (1956). The function of
cytidine coenzymes in the biosynthesis
of phospholipides. J. Biol. Chem..

[43] Ye C., Sutter B. M., Wang Y., Kuang Z., Tu B. P. (2017). A Metabolic
Function for Phospholipid and Histone Methylation. Mol. Cell.

[44] Vance J. E. (2008). Thematic
Review Series: Glycerolipids. Phosphatidylserine and Phosphatidylethanolamine
in Mammalian Cells: Two Metabolically Related Aminophospholipids. J. Lipid Res..

[45] Bartrons R., Caro J. (2007). Hypoxia, Glucose Metabolism
and the Warburg’s Effect. J. Bioenerg.
Biomembr..

