# Supplementary material for: Laser Desorption-Rapid Evaporative Ionization Mass Spectrometry (LD-REIMS): A New Tool for the High-Throughput Metabolomic and Lipidomic Profiling of Live Cells
Source: Anal Chem. 2025 Nov 27;97(48):26549–59. doi: 10.1021/acs.analchem.5c04847 (PMC12874226; doi:10.1021/acs.analchem.5c04847)
Supplement: Supplementary file 1 [file ac5c04847_si_001.pdf]

# Laser Desorption-Rapid Evaporative Ionization Mass Spectrometry (LD-REIMS): A New Tool for the High-Throughput Metabolomic and Lipidomic Profiling of Live Cells

*Stefania Maneta-Stavrakaki*[a], Aurelien Tripp[b], Daniel Simon[a], Yuchen Xiang[a], Adrienn Molnar[c], Athanasios Tsalikis[b], Efstathios Andreas Elia[d], Josephine Bunch[d], Julia Balog[e], George Poulogiannis[b], Zoltan Takats*[a]*

**AFFILIATIONS**

[a] Department of Metabolism, Digestion, and Reproduction, Imperial College London, Hammersmith Campus, Du Cane Rd, W12 0NN, London, UK

[b] Signalling and Cancer Metabolism Team, Division of Cell and Molecular Biology, The Institute of Cancer Research, 237 Fulham Rd., SW3 6JB, London, UK

[c] Hevesy György PhD School of Chemistry, ELTE Eötvös Loránd University, H-1117 Budapest, Hungary

[d] National Centre of Excellence in Mass Spectrometry Imaging (NiCE-MSI), National Physical Laboratory, Teddington, TW11 0LW, London, UK

[e] Immerse Cambridge, 301 Binney St Suite 102, Cambridge, MA 02142, United States

* Corresponding authors

# Table of Contents

**Table S1** ............................................................ S3
Table with annotations of the m/z values detected from the LD-REIMS analysis of the 5 breast and 5 colorectal cancer cell lines that were used for the validation of the method. ........................ S3
**Figure S1** ............................................................ S13
Brightfield images of live and frozen RKO cell monolayers washed with 150 mM ammonium acetate or PBS, showing intact morphology in live conditions and loss of membrane integrity following freezing. ........................ S13
**Figure S2** ............................................................ S14
Brightfield images of RKO cell monolayer before and after LD-REIMS laser ablation, showing a single ablation spot (approximately 63 µm diameter) corresponding to the sampling of approximately 10–20 cells. ........................ S14
**Table S2** ............................................................ S14
Table with the lipids validated and quantified with LC-MS. ........................ S14
**Table S3** ............................................................ S17
Table with the amino acids validated and quantified with LC-MS/MS and standard compounds. .... S17

## Table S1

Table with tentatively identified metabolites and lipids from the LD-REIMS analysis of the 5 breast and 5 colorectal cancer cell lines that were used for the validation of the method.

| Experimental *m/z* | Assignment/Annotation | Detected ion | Theroretical m/z | Error (ppm) |
|---|---|---|---|---|
| **89.024** | Lactic acid | [M-H]- | 89.0244 | -4.5 |
| **96.9596** | Sulphate | [M-H]- | 96.9601 | -5.2 |
| **99.0086** | Succinic acid | [M-H2O-H]- | 99.0088 | -2.0 |
| **99.0446** | Hydroxypentanoic acid | [M-H2O-H]- | 99.0452 | -6.1 |
| **101.0597** | Valeric acid (pentanoic acid) | [M-H]- | 101.0608 | -10.9 |
| **104.0348** | Serine | [M-H]- | 104.0353 | -4.8 |
| **111.0077** | Pentenedioic acid | [M-H2O-H]- | 111.0088 | -9.9 |
| **111.0188** | Uracil | [M-H]- | 111.02 | -10.8 |
| **113.0239** | Glutaric acid | [M-H2O-H]- | 113.0244 | -4.4 |
| **115.0031** | Malic acid | [M-H2O-H]- | 115.0037 | -5.2 |
| **115.0394** | α-ketoisovaleric acid | [M-H]- | 115.0395 | -0.9 |
| **117.0191** | Succinic acid | [M-H]- | 117.0193 | -1.7 |
| **117.0548** | Hydroxypentanoic acid | [M-H]- | 117.0557 | -7.7 |
| **123.0213** | Butyric acid | [M+Cl]- | 123.0218 | -4.1 |
| **124.0073** | Taurine | [M-H]- | 124.0074 | -0.8 |
| **127.0501** | Glutamine | [M-H2O-H]- | 127.0513 | -9.4 |
| **128.0348** | Oxoproline | [M-H]- | 128.0353 | -3.9 |
| **129.0559** | Ketoleucine | [M-H]- | 129.0557 | 1.5 |
| **129.0921** | Heptanoic acid | [M-H]- | 129.0921 | 0.0 |
| **130.0617** | Creatine | [M-H]- | 130.0622 | -3.8 |
| **130.0868** | Leucine | [M-H]- | 130.0874 | -4.6 |
| **132.947** | Phosphate | [M+Cl]- | 132.9463 | 5.3 |
| **133.0141** | Malic acid | [M-H]- | 133.0142 | -0.8 |
| **134.0471** | Adenine | [M-H]- | 134.0472 | -0.7 |
| **135.0311** | Hypoxanthine | [M-H]- | 135.0312 | -0.7 |
| **139.0161** | Hydroxybutyric acid | [M+Cl]- | 139.0167 | -4.3 |
| **140.012** | Phosphorylethanolamine | [M-H]- | 140.0118 | 1.4 |
| **141.0185** | Oxoadipic acid | [M-H2O-H]- | 141.0193 | -5.7 |
| **143.107** | Caprylic acid | [M-H]- | 143.1078 | -5.6 |
| **145.0613** | Glutamine | [M-H]- | 145.0619 | -4.1 |
| **145.0994** | Lysine | [M-H]- | 145.0983 | 7.6 |
| **146.0452** | Glutamic acid | [M-H]- | 146.0459 | -4.8 |
| **148.0446** | Methionine | [M-H]- | 148.0438 | 5.4 |
| **150.0427** | Guanine | [M-H]- | 150.0421 | 4.0 |
| **151.041** | Hydroxyphenylacetic acid | [M-H]- | 151.0401 | 6.0 |
| **152.9973** | Glycerol cyclic phosphate | [M-H]- | 152.9958 | 9.8 |
| **154.0285** | Threonine | [M+Cl]- | 154.0276 | 5.8 |
| **154.0618** | Histidine | [M-H]- | 154.0621 | -1.9 |
| **157.0146** | Ascorbic acid | [M-H2O-H]- | 157.0142 | 2.5 |
| **157.0516** | Isopropylmaleate | [M-H]- | 157.0506 | 6.4 |

| | | | | |
|---|---|---|---|---|
| **164.0711** | Phenylalanine | [M-H]- | 164.0717 | -3.7 |
| **168.0434** | Phosphorylcholine | [M-H]- | 168.0431 | 1.8 |
| **171.1385** | Decanoic acid | [M-H]- | 171.1391 | -3.5 |
| **179.0553** | Glucose | [M-H]- | 179.0561 | -4.5 |
| **180.0658** | Tyrosine | [M-H]- | 180.0666 | -4.4 |
| **181.0392** | Glutamine | [M+Cl]- | 181.0385 | 3.9 |
| **181.0713** | Mannitol/Sorbitol | [M-H]- | 181.0718 | -2.8 |
| **182.0238** | Glutamate | [M+Cl]- | 182.0226 | 6.6 |
| **184.0021** | Phosphoserine | [M-H]- | 184.0016 | 2.7 |
| **187.0014** | Xanthine | [M+Cl]- | 187.0028 | -7.5 |
| **190.0044** | Methylethanolamine phosphate | [M+Cl]- | 190.0041 | 1.6 |
| **190.052** | Hydroxyindoleacetic acid | [M-H]- | 190.051 | 5.3 |
| **195.0694** | Tryptamine | [M+Cl]- | 195.0695 | -0.5 |
| **196.0609** | Dihydroxy-L-phenylalanine | [M-H]- | 196.0615 | -3.1 |
| **199.1698** | Dodecanoic acid (FA(12:0)) | [M-H]- | 199.1704 | -3.0 |
| **200.0547** | O-Succinyl-L-homoserine | [M-H2O-H]- | 200.0564 | -8.5 |
| **203.0205** | Oxaloglutarate | [M-H]- | 203.0197 | 3.9 |
| **203.083** | Tryptophan | [M-H]- | 203.0826 | 2.0 |
| **204.0197** | Phosphodimethylethanolamine | [M+Cl]- | 204.0198 | -0.5 |
| **211.0029** | Ascorbic acid | [M+Cl]- | 211.0015 | 6.6 |
| **213.0538** | Sorbose | [M+Cl]- | 213.0535 | 1.4 |
| **214.0481** | Glucosamine | [M+Cl]- | 214.0488 | -3.3 |
| **215.0325** | Glucose | [M+Cl]- | 215.0328 | -1.4 |
| **218.0368** | Phosphocholine | [M+Cl]- | 218.0354 | 6.4 |
| **218.1029** | Pantothenic Acid | [M-H]- | 218.1034 | -2.3 |
| **225.1877** | Tetradecenoic acid (FA(14:1)) | [M-H]- | 225.186 | 7.5 |
| **227.1011** | Aspartyl-Leucine | [M-H2O-H]- | 227.1037 | -11.4 |
| **227.2011** | Tetradecanoic acid (FA(14:0)) | [M-H]- | 227.2017 | -2.6 |
| **238.0256** | Indolepyruvate | [M+Cl]- | 238.0276 | -8.4 |
| **242.079** | Cytidine | [M-H]- | 242.0782 | 3.3 |
| **243.0617** | Uridine | [M-H]- | 243.0623 | -2.5 |
| **245.0437** | Glycerophosphoglycerol | [M-H]- | 245.0432 | 2.0 |
| **253.2169** | Palmitoleic acid (FA(16:1)) | [M-H]- | 253.2173 | -1.6 |
| **255.2325** | Palmitic acid (FA(16:0)) | [M-H]- | 255.233 | -2.0 |
| **256.0601** | N-Acetyl-D-glucosamine | [M+Cl]- | 256.0593 | 3.1 |
| **266.0878** | Neuraminic acid | [M-H]- | 266.0881 | -1.1 |
| **267.0742** | Inosine | [M-H]- | 267.0735 | 2.6 |
| **271.1039** | Arginosuccinic acid | [M-H2O-H]- | 271.1048 | -3.3 |
| **277.2194** | Linolenic acid (FA(18:3)) | [M-H]- | 277.2173 | 7.6 |
| **279.0402** | Uridine | [M+Cl]- | 279.0389 | 4.7 |
| **279.2324** | Linoleic acid (FA(18:2)) | [M-H]- | 279.233 | -2.1 |
| **281.2481** | Oleic acid ((FA(18:1)) | [M-H]- | 281.2486 | -1.8 |
| **282.1576** | Histidinyl-Lysine | [M-H]- | 282.1571 | 1.8 |
| **283.107** | Glutamyl-histidine | [M-H]- | 283.1048 | 7.8 |

| | | | | |
|---|---|---|---|---|
| **283.2636** | Stearic acid (FA(18:0)) | [M-H]- | 283.2643 | -2.5 |
| **292.0708** | Glycerophosphocholine | [M+Cl]- | 292.0722 | -4.8 |
| **297.2451** | Hydroxyoctadecenoic/Oxooctadecanoic/Epoxystearic acid | [M-H]- | 297.2435 | 5.4 |
| **301.2177** | Eicosapentaenoic acid (FA(20:5)) | [M-H]- | 301.2173 | 1.3 |
| **303.2324** | Arachidonic acid (FA(20:4)) | [M-H]- | 303.233 | -2.0 |
| **305.2478** | Eicosatrienoic acid (FA(20:3)) | [M-H]- | 305.2486 | -2.6 |
| **306.0762** | Glutathione | [M-H]- | 306.0765 | -1.0 |
| **307.2643** | Eicosadienoic acid (FA(20:2)) | [M-H]- | 307.2643 | 0.0 |
| **308.0994** | N-Acetylneuraminic acid | [M-H]- | 308.0987 | 2.3 |
| **309.2797** | Eicosenoic acid (FA(20:1)) | [M-H]- | 309.2799 | -0.6 |
| **311.2968** | Arachidic acid (FA(20:0)) | [M-H]- | 311.2956 | 3.9 |
| **327.2326** | docosahexaenoic acid (DHA, FA(22:6)) | [M-H]- | 327.233 | -1.2 |
| **328.045** | Cyclic AMP | [M-H]- | 328.0452 | -0.6 |
| **329.2481** | Docosapentaenoic acid (FA(22:5)) | [M-H]- | 329.2486 | -1.5 |
| **331.2624** | Docosatetraenoic acid (FA(22:4)) | [M-H]- | 331.2643 | -5.7 |
| **333.2793** | Docosatrienoic acid (FA(22:3)) | [M-H]- | 333.2799 | -1.8 |
| **337.2352** | Eicosanoid/C20H34O4 | [M-H]- | 337.2384 | -9.5 |
| **337.3107** | Docosenoic acid (FA(22:1)) | [M-H]- | 337.3112 | -1.5 |
| **339.3264** | Docosanoic acid (FA(22:0)) | [M-H]- | 339.3269 | -1.5 |
| **341.2226** | Eicosatrienoic acid (FA(20:3)) | [M+Cl]- | 341.2253 | -7.9 |
| **346.0548** | Adenosine monophosphate (AMP) | [M-H]- | 346.0558 | -2.9 |
| **365.2097** | Trihydroxy-octadecenoic acid | [M+Cl]- | 365.21 | -0.8 |
| **365.2464** | MG(16:0) | [M+Cl]- | 365.2464 | 0.0 |
| **365.3422** | Nervonic acid (FA(24:1)) | [M-H]- | 365.3425 | -0.8 |
| **367.3574** | Lignoceric acid (FA(24:0)) | [M-H]- | 367.3582 | -2.2 |
| **389.2095** | Prostaglandin F2, H1, D1 | [M+Cl]- | 389.21 | -1.3 |
| **391.225** | LPA(16:0) | [M-H2O-H]- | 391.2255 | -1.3 |
| **391.2627** | MG(18:1) | [M+Cl]- | 391.2621 | 1.5 |
| **393.3744** | FA(26:1) | [M-H]- | 393.3738 | 1.5 |
| **395.3909** | FA(26:0) | [M-H]- | 395.3895 | 3.5 |
| **409.2363** | LPA(16:0) | [M-H]- | 409.2361 | 0.5 |
| **417.2406** | LPA(18:1) | [M-H2O-H]- | 417.2412 | -1.4 |
| **418.273** | LPE(O-16:1) | [M-H2O-H]- | 418.2729 | 0.2 |
| **419.256** | LPA(18:0) | [M-H2O-H]- | 419.2568 | -1.9 |
| **421.326** | Cholesterol | [M+Cl]- | 421.3243 | 4.0 |
| **435.2534** | LPA(18:1) | [M-H]- | 435.2517 | 3.9 |
| **436.2832** | LPE(O-16:1) | [M-H]- | 436.2833 | -0.2 |
| **437.268** | LPA(18:0) | [M-H]- | 437.2674 | 1.4 |
| **446.3046** | LPE(O-18:1) | [M-H2O-H]- | 446.3041 | 1.1 |
| **450.3001** | Hydroxyhexadecanoylcarnitine | [M+Cl]- | 450.2992 | 2.0 |
| **452.2791** | LPE(16:0) | [M-H]- | 452.2782 | 2.0 |
| **462.2992** | LPE(O-18:2) | [M-H]- | 462.299 | 0.4 |
| **464.3145** | LPE(O-18:1) | [M-H]- | 464.3146 | -0.2 |
| **465.3045** | Cholesterol sulfate | [M-H]- | 465.3044 | 0.2 |

| | | | | |
|---|---|---|---|---|
| **478.294** | LPE(18:1) | [M-H]- | 478.2939 | 0.2 |
| **480.309** | LPE(18:0) | [M-H]- | 480.3097 | -1.5 |
| **483.2737** | LPG(16:0) | [M-H]- | 483.2728 | 1.9 |
| **488.316** | LPE(20:1) | [M-H2O-H]- | 488.3146 | 2.9 |
| **491.2791** | LPG(18:1) | [M-H2O-H]- | 491.2779 | 2.4 |
| **506.3259** | LPE(20:1) | [M-H]- | 506.3252 | 1.4 |
| **508.342** | LPE(20:0) | [M-H]- | 508.3409 | 2.2 |
| **509.2891** | LPG(18:1) | [M-H]- | 509.2885 | 1.2 |
| **510.2935** | LPE(22:4) | [M-H2O-H]- | 510.299 | -10.8 |
| **524.3008** | LPS(18:0) | [M-H]- | 524.2994 | 2.7 |
| **530.3022** | LPC(16:0) | [M+Cl]- | 530.3019 | 0.6 |
| **544.4505** | Cer(d32:1) | [M+Cl]- | 544.4502 | 0.6 |
| **553.2786** | LPI(16:0) | [M-H2O-H]- | 553.2783 | 0.5 |
| **556.3167** | LPC(18:1) | [M+Cl]- | 556.3175 | -1.4 |
| **558.3277** | LPC(18:0) | [M+Cl]- | 558.3332 | -9.9 |
| **570.4658** | Cer(d34:2) | [M+Cl]- | 570.4658 | 0.0 |
| **572.4809** | Cer(d34:1) | [M+Cl]- | 572.4815 | -1.0 |
| **575.4455** | DG(30:0) | [M+Cl]- | 575.4448 | 1.2 |
| **581.3093** | LPI(18:0) | [M-H2O-H]- | 581.3096 | -0.5 |
| **598.5025** | Cer(d36:2) | [M+Cl]- | 598.4971 | 9.0 |
| **599.3207** | LPI(18:0) | [M-H]- | 599.3202 | 0.8 |
| **599.449** | DG(32:2) | [M+Cl]- | 599.4448 | 7.0 |
| **600.5129** | Cer(d36:1) | [M+Cl]- | 600.5128 | 0.2 |
| **601.4599** | DG(32:1) | [M+Cl]- | 601.4604 | -0.8 |
| **603.4739** | DG(32:0) | [M+Cl]- | 603.4761 | -3.6 |
| **627.4759** | DG(34:2) | [M+Cl]- | 627.4761 | -0.3 |
| **628.5473** | Cer(d38:1) | [M+Cl]- | 628.5441 | 5.1 |
| **629.4907** | DG(34:1) | [M+Cl]- | 629.4917 | -1.6 |
| **645.4494** | PA(32:1) | [M-H]- | 645.4501 | -1.1 |
| **647.4653** | PA(32:0) | [M-H]- | 647.4657 | -0.6 |
| **653.4912** | DG(36:3) | [M+Cl]- | 653.4917 | -0.8 |
| **654.5632** | Cer(d40:2) | [M+Cl]- | 654.5597 | 5.3 |
| **655.5064** | DG(36:2) | [M+Cl]- | 655.5074 | -1.5 |
| **656.5736** | Cer(d40:1) | [M+Cl]- | 656.5754 | -2.7 |
| **657.5197** | DG(36:1) | [M+Cl]- | 657.523 | -5.0 |
| **658.5848** | Cer(d40:0) | [M+Cl]- | 658.591 | -9.4 |
| **671.4648** | PA(34:2) | [M-H]- | 671.4657 | -1.3 |
| **672.497** | PE(O-32:2) | [M-H]- | 672.4974 | -0.6 |
| **673.481** | PA(34:1) | [M-H]- | 673.4814 | -0.6 |
| **677.4924** | DG(38:5) | [M+Cl]- | 677.4917 | 1.0 |
| **679.5057** | DG(38:4) | [M+Cl]- | 679.5074 | -2.5 |
| **680.573** | Cer(d42:3) | [M+Cl]- | 680.5754 | -3.5 |
| **682.5896** | Cer(d42:2) | [M+Cl]- | 682.591 | -2.1 |
| **683.5435** | DG(38:2) | [M+Cl]- | 683.5387 | 7.0 |

| | | | | |
|---|---|---|---|---|
| **684.6037** | Cer(d42:1) | [M+Cl]- | 684.6067 | -4.4 |
| **686.4794** | PE(32:2) | [M-H]- | 686.4766 | 4.1 |
| **688.4924** | PE(32:1) | [M-H]- | 688.4923 | 0.1 |
| **689.4963** | DG(38:6) | [M+Cl]- | 689.4917 | 6.7 |
| **690.5071** | PE(32:0) | [M-H]- | 690.5079 | -1.2 |
| **693.4727** | PG(30:0) | [M-H]- | 693.4712 | 2.2 |
| **697.4809** | PA(36:3) | [M-H]- | 697.4814 | -0.7 |
| **698.5106** | PE(O-34:3) | [M-H]- | 698.513 | -3.4 |
| **699.4965** | PA(36:2) | [M-H]- | 699.497 | -0.7 |
| **700.5272** | PE(O-34:2) | [M-H]- | 700.5287 | -2.1 |
| **702.5398** | PE(O-34:1) | [M-H]- | 702.5443 | -6.4 |
| **703.5131** | DG(40:6) | [M+Cl]- | 703.5074 | 8.1 |
| **705.5245** | DG(40:5) | [M+Cl]- | 705.523 | 2.1 |
| **709.5047** | SM(d32:1) | [M+Cl]- | 709.5057 | -1.4 |
| **714.5068** | PE(34:2) | [M-H]- | 714.5079 | -1.5 |
| **716.5233** | PE(34:1) | [M-H]- | 716.5236 | -0.4 |
| **718.5363** | PE(34:0) | [M-H]- | 718.5392 | -4.0 |
| **719.4856** | PG(32:1) | [M-H]- | 719.4869 | -1.8 |
| **720.4954** | PE(O-36:6) | [M-H]- | 720.4974 | -2.8 |
| **721.4989** | PG(32:0) | [M-H]- | 721.5025 | -5.0 |
| **722.5128** | PE(O-36:5) | [M-H]- | 722.513 | -0.3 |
| **725.5143** | PA(38:3) | [M-H]- | 725.5127 | 2.2 |
| **727.5315** | PA(38:2) | [M-H]- | 727.5283 | 4.4 |
| **728.559** | PE(O-36:2) | [M-H]- | 728.56 | -1.4 |
| **729.5285** | DG(42:7) | [M+Cl]- | 729.523 | 7.5 |
| **730.573** | PE(O-36:1) | [M-H]- | 730.5756 | -3.6 |
| **731.5421** | DG(42:6) | [M+Cl]- | 731.5387 | 4.6 |
| **734.5317** | GlcCer(d34:1) | [M+Cl]- | 734.5343 | -3.5 |
| **736.4902** | PE(36:5) | [M-H]- | 736.4923 | -2.9 |
| **737.4882** | PA(36:1) | [M+Cl]- | 737.4894 | -1.6 |
| **737.5357** | SM(d34:1) | [M+Cl]- | 737.537 | -1.8 |
| **738.5081** | PE(36:4) | [M-H]- | 738.5079 | 0.3 |
| **740.5039** | PC(30:0) | [M+Cl]- | 740.5003 | 4.9 |
| **742.5387** | PE(36:2) | [M-H]- | 742.5392 | -0.7 |
| **744.5538** | PE(36:1) | [M-H]- | 744.5549 | -1.5 |
| **745.5004** | PG(34:2) | [M-H]- | 745.5025 | -2.8 |
| **746.5113** | PE(O-38:7) | [M-H]- | 746.513 | -2.3 |
| **746.5625** | PE(36:0) | [M-H]- | 746.5705 | -10.7 |
| **747.517** | PG(34:1) | [M-H]- | 747.5182 | -1.6 |
| **748.525** | PE(O-38:6) | [M-H]- | 748.5287 | -4.9 |
| **749.5299** | PG(34:0) | [M-H]- | 749.5338 | -5.2 |
| **750.5413** | PE(O-38:5) | [M-H]- | 750.5443 | -4.0 |
| **752.495** | PE(34:1) | [M+Cl]- | 752.5003 | -7.0 |
| **757.5564** | DG(44:7) | [M+Cl]- | 757.5543 | 2.8 |

| | | | | |
|---|---|---|---|---|
| **758.49** | PE(O-36:5) | [M+Cl]- | 758.4897 | 0.4 |
| **759.5718** | DG(44:6) | [M+Cl]- | 759.57 | 2.4 |
| **760.5146** | PS(34:1) | [M-H]- | 760.5134 | 1.6 |
| **761.5805** | DG(44:5) | [M+Cl]- | 761.5856 | -6.7 |
| **763.549** | SM(d36:2) | [M+Cl]- | 763.5526 | -4.7 |
| **764.5237** | PE(38:5) | [M-H]- | 764.5236 | 0.1 |
| **766.5315** | PE(38:4) | [M-H]- | 766.5392 | -10.0 |
| **770.5679** | PE(38:2) | [M-H]- | 770.5705 | -3.4 |
| **772.5266** | PE(O-40:8) | [M-H]- | 772.5287 | -2.7 |
| **772.584** | PE(38:1) | [M-H]- | 772.5862 | -2.8 |
| **773.5334** | PG(36:2) | [M-H]- | 773.5338 | -0.5 |
| **774.5952** | PE(38:0) | [M-H]- | 774.6018 | -8.5 |
| **775.5474** | PG(36:1) | [M-H]- | 775.5495 | -2.7 |
| **776.5572** | PE(O-40:6) | [M-H]- | 776.56 | -3.6 |
| **777.5624** | PG(36:0) | [M-H]- | 777.5651 | -3.5 |
| **778.5125** | PE(36:2) | [M+Cl]- | 778.5159 | -4.4 |
| **778.5705** | PE(O-40:5) | [M-H]- | 778.5756 | -6.6 |
| **780.5341** | PE(36:1) | [M+Cl]- | 780.5316 | 3.2 |
| **786.5285** | PS(36:2) | [M-H]- | 786.5291 | -0.8 |
| **788.5437** | PS(36:1) | [M-H]- | 788.5447 | -1.3 |
| **790.5505** | PE(O-38:3) | [M+Cl]- | 790.5523 | -2.3 |
| **791.4702** | PA(44:12) | [M-H]- | 791.4657 | 5.7 |
| **791.5072** | PI(32:0) | [M-H2O-H]- | 791.508 | -1.0 |
| **791.589** | SM(d38:2) | [M+Cl]- | 791.5839 | 6.4 |
| **793.5013** | PG(38:6) | [M-H]- | 793.5025 | -1.5 |
| **794.548** | PC(34:1) | [M+Cl]- | 794.5472 | 1.0 |
| **798.5984** | PE(40:2) | [M-H]- | 798.6018 | -4.3 |
| **800.4989** | PE(38:5) | [M+Cl]- | 800.5003 | -1.7 |
| **800.6159** | PE(40:1) | [M-H]- | 800.6175 | -2.0 |
| **801.55** | PA(44:7) | [M-H]- | 801.544 | 7.5 |
| **802.5106** | PE(38:4) | [M+Cl]- | 802.5159 | -6.6 |
| **804.5753** | PC(O-36:3) | [M+Cl]- | 804.5679 | 9.2 |
| **805.5784** | PA(44:5) | [M-H]- | 805.5753 | 3.8 |
| **805.6084** | TG(46:4) | [M+Cl]- | 805.6118 | -4.2 |
| **806.5003** | PS(38:6) | [M-H]- | 806.4977 | 3.2 |
| **806.5509** | PE(38:2) | [M+Cl]- | 806.5472 | 4.6 |
| **806.5878** | PC(O-36:2) | [M+Cl]- | 806.5836 | 5.2 |
| **807.5902** | PA(44:4) | [M-H]- | 807.5909 | -0.9 |
| **808.5119** | PS(38:5) | [M-H]- | 808.5134 | -1.9 |
| **808.5642** | PE(38:1) | [M+Cl]- | 808.5629 | 1.6 |
| **808.5974** | PC(O-36:1) | [M+Cl]- | 808.5992 | -2.2 |
| **809.5178** | PI(32:0) | [M-H]- | 809.5186 | -1.0 |
| **810.5286** | PS(38:4) | [M-H]- | 810.5291 | -0.6 |
| **812.542** | PS(38:3) | [M-H]- | 812.5447 | -3.3 |

| | | | | |
|---|---|---|---|---|
| **813.546** | PG(36:0) | [M+Cl]- | 813.5418 | 5.2 |
| **814.5164** | PC(36:5) | [M+Cl]- | 814.5159 | 0.6 |
| **815.4904** | PG and/or BMP(40:9) | [M-H]- | 815.4869 | 4.3 |
| **815.5776** | SM(d40:3) | [M+Cl]- | 815.5839 | -7.7 |
| **816.5314** | PC(36:4) | [M+Cl]- | 816.5316 | -0.2 |
| **816.5725** | PS(38:1) | [M-H]- | 816.576 | -4.3 |
| **817.5042** | PG and/or BMP(40:8) | [M-H]- | 817.5025 | 2.1 |
| **818.5461** | PC(36:3) | [M+Cl]- | 818.5472 | -1.3 |
| **818.5892** | PS(38:0) | [M-H]- | 818.5917 | -3.1 |
| **819.5185** | PG and/or BMP(40:7) | [M-H]- | 819.5182 | 0.4 |
| **820.5626** | PC(36:2) | [M+Cl]- | 820.5629 | -0.4 |
| **821.5318** | PG and/or BMP(40:6) | [M-H]- | 821.5338 | -2.4 |
| **821.6258** | SM(d40:1) | [M+Cl]- | 821.6309 | -6.2 |
| **822.5728** | PC(36:1) | [M+Cl]- | 822.5785 | -6.9 |
| **826.5551** | PC(O-38:6) | [M+Cl]- | 826.5523 | 3.4 |
| **826.6291** | PE(42:2) | [M-H]- | 826.6331 | -4.8 |
| **828.4853** | PS(40:9) | [M-H]- | 828.4821 | 3.9 |
| **828.5359** | PE(40:5) | [M+Cl]- | 828.5316 | 5.2 |
| **828.646** | PE(42:1) | [M-H]- | 828.6488 | -3.4 |
| **832.5958** | PC(O-38:3) | [M+Cl]- | 832.5992 | -4.1 |
| **834.5261** | PS(40:6) | [M-H]- | 834.5291 | -3.6 |
| **834.585** | PE(40:2) | [M+Cl]- | 834.5785 | 7.8 |
| **835.5314** | PI(34:1) | [M-H]- | 835.5342 | -3.4 |
| **836.5391** | PS(40:5) | [M-H]- | 836.5447 | -6.7 |
| **838.4419** | PS(38:8) | [M+Cl]- | 838.4431 | -1.4 |
| **838.6423** | PC(O-38:0) | [M+Cl]- | 838.6462 | -4.7 |
| **839.6894** | TG(48:1) | [M+Cl]- | 839.6901 | -0.8 |
| **840.4544** | PS(38:7) | [M+Cl]- | 840.4588 | -5.2 |
| **842.5443** | PC(38:5) | [M+Cl]- | 842.5467 | -2.8 |
| **842.5875** | PS(40:2) | [M-H]- | 842.5917 | -5.0 |
| **843.5176** | PG and/or BMP(42:9) | [M-H]- | 843.5182 | -0.7 |
| **844.4655** | PE(42:11) | [M+Cl]- | 844.469 | -4.1 |
| **844.6053** | PS(40:1) | [M-H]- | 844.6073 | -2.4 |
| **844.6413** | GlcCer(d42:2) | [M+Cl]- | 844.6439 | -3.1 |
| **845.533** | PG and/or BMP(42:8) | [M-H]- | 845.5338 | -0.9 |
| **846.5733** | PC(38:3) | [M+Cl]- | 846.5785 | -6.1 |
| **846.6551** | GlcCer(d42:1) | [M+Cl]- | 846.6595 | -5.2 |
| **847.5984** | PA(44:2) | [M+Cl]- | 847.5989 | -0.6 |
| **847.6445** | SM(d42:2) | [M+Cl]- | 847.6465 | -2.4 |
| **848.6005** | PC(38:2) | [M+Cl]- | 848.5942 | 7.4 |
| **849.6547** | SM(d40:1) | [M+Cl]- | 849.6622 | -8.8 |
| **850.5535** | PC(O-40:8) | [M+Cl]- | 850.5523 | 1.4 |
| **850.6337** | PE(44:4) | [M-H]- | 850.6331 | 0.7 |
| **851.5939** | TG(50:9) | [M+Cl]- | 851.5962 | -2.7 |

| | | | | |
|---|---|---|---|---|
| **852.5714** | PC(O-40:7) | [M+Cl]- | 852.5679 | 4.1 |
| **852.624** | PC(38:0) | [M+Cl]- | 852.6255 | -1.8 |
| **852.6478** | PE(44:3) | [M-H]- | 852.6488 | -1.2 |
| **853.611** | TG(50:8) | [M+Cl]- | 853.6118 | -0.9 |
| **854.5716** | PS(38:0) | [M+Cl]- | 854.5683 | 3.9 |
| **854.5814** | PC(O-40:6) | [M+Cl]- | 854.5836 | -2.6 |
| **857.5174** | PI(36:4) | [M-H]- | 857.5186 | -1.4 |
| **858.5753** | PE(42:4) | [M+Cl]- | 858.5785 | -3.7 |
| **859.5325** | PI(36:3) | [M-H]- | 859.5342 | -2.0 |
| **860.6378** | PC(O-40:3) | [M+Cl]- | 860.6305 | 8.5 |
| **861.5493** | PI(36:2) | [M-H]- | 861.5499 | -0.7 |
| **862.6511** | PC(O-40:2) | [M+Cl]- | 862.6462 | 5.7 |
| **863.5638** | PI(36:1) | [M-H]- | 863.5655 | -2.0 |
| **864.6192** | PE(42:1) | [M+Cl]- | 864.6255 | -7.3 |
| **864.6589** | PC(O-40:1) | [M+Cl]- | 864.6618 | -3.4 |
| **865.501** | PG and/or BMP(44:12) | [M-H]- | 865.5025 | -1.7 |
| **866.5884** | PS(42:4) | [M-H]- | 866.5917 | -3.8 |
| **867.5149** | PG and/or BMP(44:11) | [M-H]- | 867.5182 | -3.8 |
| **867.7239** | TG(50:1) | [M+Cl]- | 867.7214 | 2.9 |
| **868.6037** | PS(42:3) | [M-H]- | 868.6073 | -4.1 |
| **870.5753** | PC(40:5) | [M+Cl]- | 870.5785 | -3.7 |
| **872.5963** | PC(40:4) | [M+Cl]- | 872.5942 | 2.4 |
| **872.6793** | GlcCer(d44:2) | [M+Cl]- | 872.6752 | 4.7 |
| **874.5215** | PE(44:10) | [M+Cl]- | 874.5159 | 6.4 |
| **874.6157** | PC(40:3) | [M+Cl]- | 874.6098 | 6.7 |
| **874.6874** | GlcCer(d44:1) | [M+Cl]- | 874.6908 | -3.9 |
| **875.6753** | SM(d44:2) | [M+Cl]- | 875.6778 | -2.9 |
| **876.5267** | PE(44:9) | [M+Cl]- | 876.5316 | -5.6 |
| **876.5598** | PS(40:3) | [M+Cl]- | 876.5527 | 8.1 |
| **879.6294** | TG(52:9) | [M+Cl]- | 879.6275 | 2.2 |
| **881.5218** | PI(38:6) | [M-H]- | 881.5186 | 3.6 |
| **883.5335** | PI(38:5) | [M-H]- | 883.5342 | -0.8 |
| **885.5493** | PI(38:4) | [M-H]- | 885.5499 | -0.7 |
| **887.5618** | PI(38:3) | [M-H]- | 887.5655 | -4.2 |
| **888.6615** | PC(O-42:3) | [M+Cl]- | 888.6618 | -0.3 |
| **890.5543** | PC(42:9) | [M+Cl]- | 890.5472 | 8.0 |
| **891.7143** | TG(52:3) | [M+Cl]- | 891.7214 | -8.0 |
| **893.7352** | TG(52:2) | [M+Cl]- | 893.737 | -2.0 |
| **895.7544** | TG(52:1) | [M+Cl]- | 895.7527 | 1.9 |
| **896.5834** | LacCer(d34:1) | [M+Cl]- | 896.5871 | -4.1 |
| **897.5226** | PI(36:2) | [M+Cl]- | 897.5265 | -4.3 |
| **905.518** | PG and/or BMP(44:10) | [M+Cl]- | 905.5105 | 8.3 |
| **906.6002** | PS(42:2) | [M+Cl]- | 906.5996 | 0.7 |
| **907.6574** | TG(54:9) | [M+Cl]- | 907.6588 | -1.5 |

| | | | | |
|---|---|---|---|---|
| **911.5659** | PI(40:5) | [M-H]- | 911.5655 | 0.4 |
| **913.5791** | PI(40:4) | [M-H]- | 913.5812 | -2.3 |
| **916.568** | PC(44:10) | [M+Cl]- | 916.5629 | 5.6 |
| **917.7348** | TG(54:4) | [M+Cl]- | 917.737 | -2.4 |
| **919.746** | TG(54:3) | [M+Cl]- | 919.7527 | -7.3 |
| **921.7629** | TG(54:2) | [M+Cl]- | 921.7683 | -5.9 |
| **922.6016** | LacCer(d36:2) | [M+Cl]- | 922.6028 | -1.3 |
| **922.6488** | PS(46:4) | [M-H]- | 922.6543 | -6.0 |
| **923.7803** | TG(54:1) | [M+Cl]- | 923.784 | -4.0 |
| **939.7181** | TG(56:7) | [M+Cl]- | 939.7214 | -3.5 |
| **945.7663** | TG(56:4) | [M+Cl]- | 945.7683 | -2.1 |
| **1021.4859** | PIP2(36:2) | [M-H]- | 1021.4825 | 3.3 |
| **1023.4916** | PIP2(36:1) | [M-H]- | 1023.4982 | -6.4 |
| **1053.8617** | TG(64:6) | [M+Cl]- | 1053.8622 | -0.5 |
| **1057.8826** | TG(64:4) | [M+Cl]- | 1057.8935 | -10.3 |
| **1063.9472** | TG(64:1) | [M+Cl]- | 1063.9405 | 6.3 |
| **1289.7426** | Ganglioside GA1 (d36:1) | [M+Cl]- | 1289.7506 | -6.2 |
| **1347.9011** | Ganglioside GA1 (d44:1) | [M-H2O-H]- | 1347.8886 | 9.3 |
| **1355.8077** | Ganglioside GM2 (d34:0) | [M-H]- | 1355.8057 | 1.5 |
| **1382.8147** | Ganglioside GM2 (d36:1) | [M-H]- | 1382.8166 | -1.4 |
| **1401.992** | CL(68:3) | [M-H]- | 1401.9806 | 8.1 |
| **1423.9518** | CL(70:6) | [M-H]- | 1423.965 | -9.3 |
| **1425.9207** | CL(72:10) | [M-H2O-H]- | 1425.9231 | -1.7 |
| **1425.9809** | CL(70:5) | [M-H]- | 1425.9806 | 0.2 |
| **1428.0012** | CL(70:4) | [M-H]- | 1427.9963 | 3.4 |
| **1430.0178** | CL(70:3) | [M-H]- | 1430.0119 | 4.1 |
| **1442.0394** | CL(72:2) | [M-H2O-H]- | 1442.0483 | -6.2 |
| **1442.7956** | Ganglioside GD3 (d34:1) | [M-H]- | 1442.8013 | -4.0 |
| **1443.9406** | CL(72:4) | [M-H]- | 1443.9337 | 4.8 |
| **1451.8898** | CL(70:10) | [M+Cl]- | 1451.879 | 7.4 |
| **1454.0029** | CL(72:5) | [M-H]- | 1454.0119 | -6.2 |
| **1456.017** | CL(72:4) | [M-H]- | 1456.027 | -6.9 |
| **1460.0489** | CL(72:2) | [M-H]- | 1460.0589 | -6.8 |
| **1462.0676** | CL(72:1) | [M-H]- | 1462.0745 | -4.7 |
| **1463.8949** | CL(74:14) | [M-H]- | 1463.9023 | -5.1 |
| **1464.0813** | CL(72:0) | [M-H]- | 1464.0902 | -6.1 |
| **1465.9963** | CL(70:3) | [M+Cl]- | 1465.9886 | 5.3 |
| **1466.0408** | CL(74:4) | [M-H2O-H]- | 1466.0483 | -5.1 |
| **1470.013** | CL(70:1) | [M+Cl]- | 1470.0199 | -4.7 |
| **1471.9729** | CL(74:10) | [M-H]- | 1471.965 | 5.4 |
| **1480.0341** | CL(74:6) | [M-H]- | 1480.0276 | 4.4 |
| **1487.8087** | Ganglioside GM1 (d32:1) | [M-H]- | 1487.8115 | -1.9 |
| **1488.0284** | CL(76:7) | [M-H2O-H]- | 1488.0326 | -2.8 |
| **1488.0854** | CL(74:2) | [M-H]- | 1488.0902 | -3.2 |

| | | | | |
|---|---|---|---|---|
| **1491.9188** | CL(76:14) | [M-H]- | 1491.9337 | -10.0 |
| **1492.0032** | CL(72:4) | [M+Cl]- | 1492.0042 | -0.7 |
| **1492.0617** | CL(76:5) | [M-H2O-H]- | 1492.0639 | -1.5 |
| **1494.0168** | CL(72:3) | [M+Cl]- | 1494.0199 | -2.1 |
| **1494.0761** | CL(76:4) | [M-H2O-H]- | 1494.0796 | -2.3 |
| **1496.0406** | CL(72:2) | [M+Cl]- | 1496.0355 | 3.4 |
| **1498.0466** | CL(72:1) | [M+Cl]- | 1498.0512 | -3.1 |
| **1498.1172** | CL(76:2) | [M-H2O-H]- | 1498.1109 | 4.2 |
| **1498.818** | Ganglioside GM1 (34:1) | [M-H2O-H]- | 1498.8275 | -6.3 |
| **1500.0721** | CL(72:0) | [M+Cl]- | 1500.0668 | 3.5 |

## Figure S1

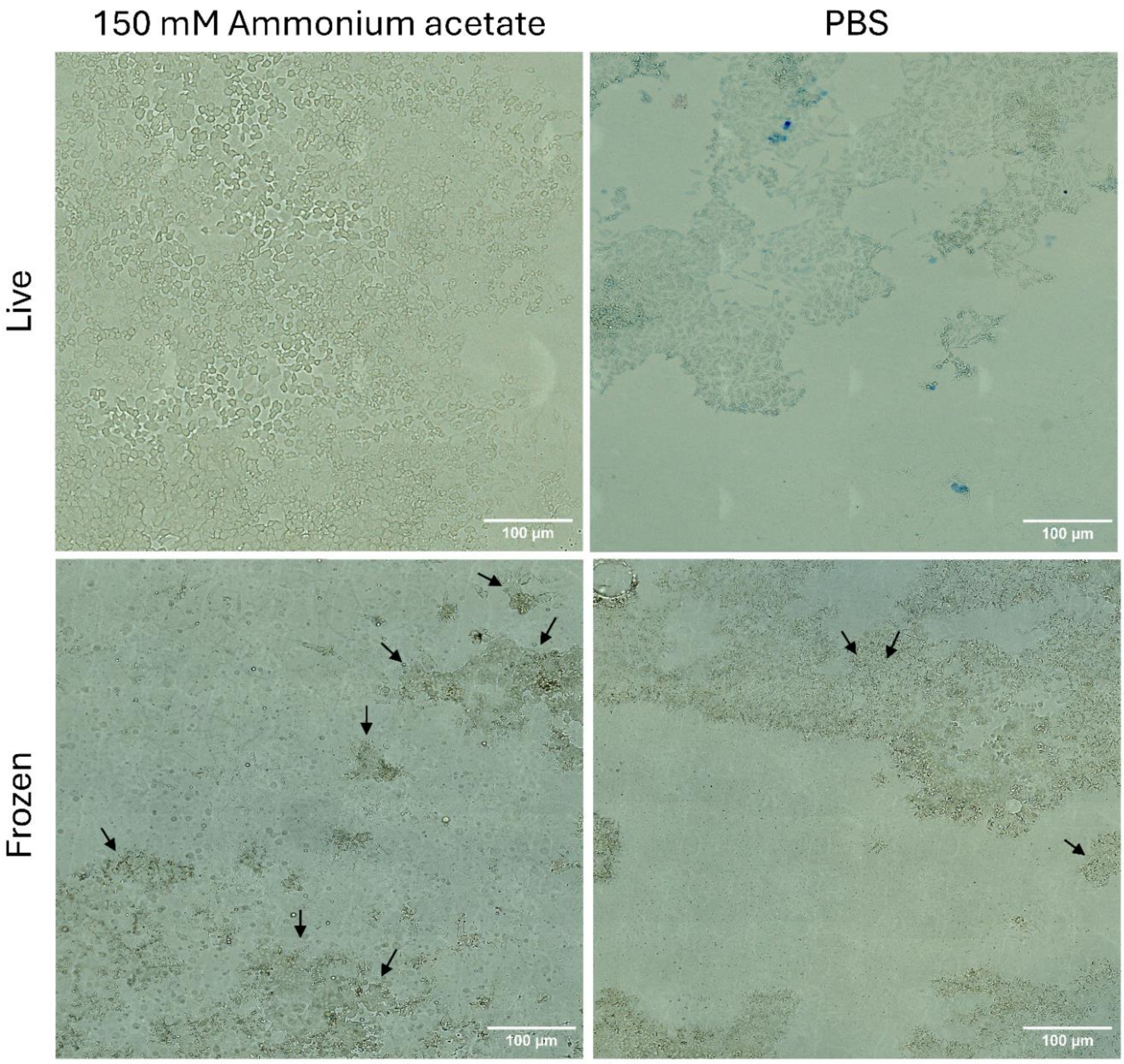


**Figure S1.** Brightfield microscope images of live (top) and frozen (bottom) RKO cell monolayers washed twice with 150 mM ammonium acetate (left) or PBS (right). Live cell monolayers, regardless of the washing solution, display a homogeneous and intact cell population. In contrast, frozen cells show evident loss of membrane integrity, cellular debris, and surface artefacts (some indicated by arrows), consistent with cell rupture and loss of structural integrity following freezing and thawing.

## Figure S2

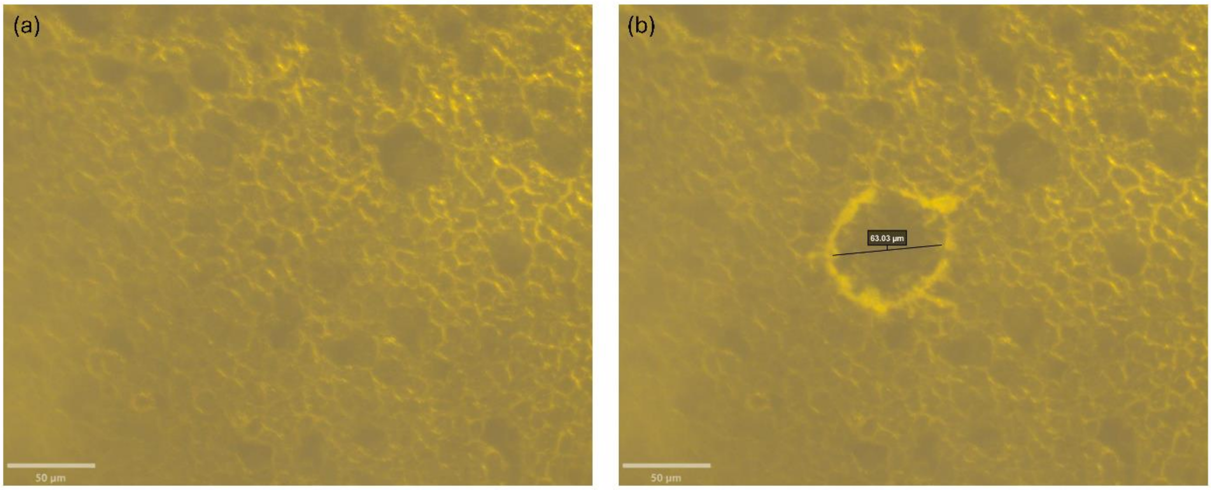


**Figure S2.** Brightfield images of an RKO cell monolayer (a) before and (b) after laser ablation with LD-REIMS. The ablation spot has an approximate diameter of 63 µm, as shown in (b). This corresponds to a sampled area of approximately 3.1 × $10^3$ µm$^2$, representing approximately 10–20 cells depending on cell size and local confluence. Scale bar = 50 µm.

## Table S2

Table with the lipids validated and quantified with LC-MS.

| | LD-REIMS | | LC-MS | | |
|---|---|---|---|---|---|
| **LipidID** | **m/z** | **Detected ion** | **Retention time (min)** | **m/z** | **Detected ion** |
| **FA(14:0)** | 227.2011 | [M-H]- | 2.22 | 227.201 | [M-H]- |
| **FA(16:1)** | 253.2169 | [M-H]- | 2.37 | 253.2168 | [M-H]- |
| **FA(16:0)** | 255.2325 | [M-H]- | 2.81 | 255.2323 | [M-H]- |
| **FA(18:3)** | 277.2194 | [M-H]- | 2.24 | 277.2163 | [M-H]- |
| **FA(18:2)** | 279.2324 | [M-H]- | 2.52 | 279.2322 | [M-H]- |
| **FA(18:1)** | 281.2481 | [M-H]- | 2.96 | 281.2479 | [M-H]- |
| **FA(18:0)** | 283.2636 | [M-H]- | 3.51 | 283.2632 | [M-H]- |
| **FA(20:4)** | 303.2324 | [M-H]- | 2.48 | 303.2323 | [M-H]- |
| **FA(20:3)** | 305.2478 | [M-H]- | 2.73 | 305.2477 | [M-H]- |
| **FA(20:2)** | 307.2643 | [M-H]- | 3.13 | 307.2635 | [M-H]- |
| **FA(20:1)** | 309.2797 | [M-H]- | 3.63 | 309.2794 | [M-H]- |
| **FA(20:0)** | 311.2968 | [M-H]- | 4.3 | 311.2947 | [M-H]- |
| **FA(22:6)** | 327.2326 | [M-H]- | 2.37 | 327.2166 | [M-H]- |
| **FA(22:5)** | 329.2481 | [M-H]- | 2.71 | 329.2475 | [M-H]- |
| **FA(22:4)** | 331.2624 | [M-H]- | 2.94 | 331.2631 | [M-H]- |
| **FA(22:3)** | 333.2793 | [M-H]- | 3.4 | 333.2785 | [M-H]- |
| **FA(22:1)** | 337.3107 | [M-H]- | 4.37 | 337.3107 | [M-H]- |
| **FA(22:0)** | 339.3264 | [M-H]- | 5.13 | 339.3262 | [M-H]- |
| **FA(24:1)** | 365.3422 | [M-H]- | 5.16 | 365.3414 | [M-H]- |
| **FA(24:0)** | 367.3574 | [M-H]- | 5.96 | 367.3571 | [M-H]- |
| **FA(26:1)** | 393.3744 | [M-H]- | 5.97 | 393.3727 | [M-H]- |

| | | | | | |
|---|---|---|---|---|---|
| **FA(26:0)** | 395.3909 | [M-H]- | 6.75 | 395.3882 | [M-H]- |
| **PI(32:0)** | 809.5178 | [M-H]- | 5.35 | 809.5166 | [M-H]- |
| **PI(34:1)** | 835.5314 | [M-H]- | 5.53 | 835.533 | [M-H]- |
| **PI(36:4)** | 857.5174 | [M-H]- | 5.04 | 857.5159 | [M-H]- |
| **PI(36:3)** | 859.5325 | [M-H]- | 5.21 | 859.5301 | [M-H]- |
| **PI(36:2)** | 861.5493 | [M-H]- | 5.65 | 861.5471 | [M-H]- |
| **PI(36:1)** | 863.5638 | [M-H]- | 6.17 | 863.5614 | [M-H]- |
| **PI(38:6)** | 881.5218 | [M-H]- | 4.88 | 881.5168 | [M-H]- |
| **PI(38:5)** | 883.5335 | [M-H]- | 5.16 | 883.5309 | [M-H]- |
| **PI(38:4)** | 885.5493 | [M-H]- | 5.71 | 885.5478 | [M-H]- |
| **PI(38:3)** | 887.5618 | [M-H]- | 5.89 | 887.562 | [M-H]- |
| **PI(40:5)** | 911.5659 | [M-H]- | 5.68 | 911.5607 | [M-H]- |
| **PI(40:4)** | 913.5791 | [M-H]- | 6.1 | 913.5768 | [M-H]- |
| **PS(34:1)** | 760.5146 | [M-H]- | 5.56 | 760.5137 | [M-H]- |
| **PS(36:2)** | 786.5285 | [M-H]- | 5.68 | 786.518 | [M-H]- |
| **PS(36:1)** | 788.5437 | [M-H]- | 6.2 | 788.5446 | [M-H]- |
| **PS(38:6)** | 806.5003 | [M-H]- | 4.39 | 806.5068 | [M-H]- |
| **PS(38:5)** | 808.5119 | [M-H]- | 4.88 | 808.5068 | [M-H]- |
| **PS(38:4)** | 810.5286 | [M-H]- | 5.73 | 810.5278 | [M-H]- |
| **PS(38:3)** | 812.542 | [M-H]- | 5.92 | 812.536 | [M-H]- |
| **PS(38:1)** | 816.5725 | [M-H]- | 6.01 | 816.5993 | [M-H]- |
| **PS(38:0)** | 818.5892 | [M-H]- | 6.52 | 818.5891 | [M-H]- |
| **PS(40:5)** | 836.5391 | [M-H]- | 5.53 | 836.538 | [M-H]- |
| **PS(40:2)** | 842.5875 | [M-H]- | 6.88 | 842.5764 | [M-H]- |
| **PS(40:1)** | 844.6053 | [M-H]- | 7.44 | 844.6007 | [M-H]- |
| **PS(42:3)** | 868.6037 | [M-H]- | 7.02 | 868.5895 | [M-H]- |
| **PE(32:0)** | 690.5071 | [M-H]- | 5.33 | 692.5245 | [M+H]+ |
| **PE(34:2)** | 714.5068 | [M-H]- | 6.19 | 716.5475 | [M+H]+ |
| **PE(34:1)** | 716.5233 | [M-H]- | 6.73 | 718.5566 | [M+H]+ |
| **PE(34:0)** | 718.5363 | [M-H]- | 6.79 | 720.5781 | [M+H]+ |
| **PE(36:5)** | 736.4902 | [M-H]- | 6.86 | 738.5444 | [M+H]+ |
| **PE(36:4)** | 738.5081 | [M-H]- | 6.16 | 740.5356 | [M+H]+ |
| **PE(36:2)** | 742.5387 | [M-H]- | 6.84 | 744.5751 | [M+H]+ |
| **PE(36:1)** | 744.5538 | [M-H]- | 7.34 | 746.5822 | [M+H]+ |
| **PE(36:0)** | 746.5625 | [M-H]- | 7.43 | 748.6065 | [M+H]+ |
| **PE(38:5)** | 764.5237 | [M-H]- | 6.31 | 766.5522 | [M+H]+ |
| **PE(38:4)** | 766.5315 | [M-H]- | 6.84 | 768.5681 | [M+H]+ |
| **PE(38:2)** | 770.5679 | [M-H]- | 7.5 | 772.6091 | [M+H]+ |
| **PE(40:2)** | 798.5984 | [M-H]- | 7.97 | 800.6317 | [M+H]+ |
| **PE(40:1)** | 800.6159 | [M-H]- | 8.5 | 802.6359 | [M+H]+ |
| **PE(42:2)** | 826.6291 | [M-H]- | 8.47 | 828.6548 | [M+H]+ |
| **PE(42:1)** | 828.646 | [M-H]- | 9.01 | 830.6638 | [M+H]+ |
| **LPE(16:0)** | 452.2791 | [M-H]- | 1.94 | 454.2929 | [M+H]+ |
| **LPE(18:1)** | 478.294 | [M-H]- | 2.1 | 480.3225 | [M+H]+ |

| | | | | | |
|---|---|---|---|---|---|
| **LPE(18:0)** | 480.309 | [M-H]- | 2.49 | 482.3879 | [M+H]+ |
| **LPE(20:1)** | 506.3259 | [M-H]- | 2.23 | 508.3763 | [M+H]+ |
| **LPE(20:0)** | 508.342 | [M-H]- | 2.67 | 510.3909 | [M+H]+ |
| **LPE(O-18:2)** | 462.2992 | [M-H]- | 2.31 | 464.3139 | [M+H]+ |
| **LPE(O-18:1)** | 464.3145 | [M-H]- | 2.77 | 466.3296 | [M+H]+ |
| **PC(30:0)** | 740.5039 | [M+Cl]- | 5.69 | 706.5486 | [M+H]+ |
| **PC(34:1)** | 794.548 | [M+Cl]- | 6.53 | 760.5864 | [M+H]+ |
| **PC(36:5)** | 814.5164 | [M+Cl]- | 5.51 | 780.5536 | [M+H]+ |
| **PC(36:4)** | 816.5314 | [M+Cl]- | 6.01 | 782.5688 | [M+H]+ |
| **PC(36:3)** | 818.5461 | [M+Cl]- | 6.17 | 784.585 | [M+H]+ |
| **PC(36:2)** | 820.5626 | [M+Cl]- | 6.66 | 786.6004 | [M+H]+ |
| **PC(36:1)** | 822.5728 | [M+Cl]- | 7.18 | 788.6238 | [M+H]+ |
| **PC(38:5)** | 842.5443 | [M+Cl]- | 6.14 | 808.5843 | [M+H]+ |
| **PC(38:3)** | 846.5733 | [M+Cl]- | 6.83 | 812.615 | [M+H]+ |
| **PC(38:2)** | 848.6005 | [M+Cl]- | 7.24 | 814.6387 | [M+H]+ |
| **PC(40:5)** | 870.5753 | [M+Cl]- | 6.71 | 836.6166 | [M+H]+ |
| **PC(O-36:3)** | 804.5753 | [M+Cl]- | 6.63 | 770.5926 | [M+H]+ |
| **PC(O-36:2)** | 806.5878 | [M+Cl]- | 7.04 | 772.6169 | [M+H]+ |
| **PC(O-36:1)** | 808.5974 | [M+Cl]- | 7.58 | 774.6328 | [M+H]+ |
| **PC(O-38:6)** | 826.5551 | [M+Cl]- | 6.23 | 792.5809 | [M+H]+ |
| **PC(O-38:3)** | 832.5958 | [M+Cl]- | 7.29 | 798.6266 | [M+H]+ |
| **PC(O-38:0)** | 838.6423 | [M+Cl]- | 8.6 | 804.6825 | [M+H]+ |
| **PC(O-40:7)** | 852.5714 | [M+Cl]- | 6.32 | 818.6057 | [M+H]+ |
| **PC(O-40:6)** | 854.5814 | [M+Cl]- | 6.89 | 820.6171 | [M+H]+ |
| **PC(O-40:3)** | 860.6378 | [M+Cl]- | 7.86 | 826.6698 | [M+H]+ |
| **PC(O-40:2)** | 862.6511 | [M+Cl]- | 8.18 | 828.6868 | [M+H]+ |
| **PC(O-40:1)** | 864.6589 | [M+Cl]- | 8.59 | 830.7006 | [M+H]+ |
| **PC(O-42:3)** | 888.6615 | [M+Cl]- | 8.28 | 854.6967 | [M+H]+ |
| **Cer(d34:2)** | 570.4658 | [M+Cl]- | 5.75 | 536.5044 | [M+H]+ |
| **Cer(d34:1)** | 572.4809 | [M+Cl]- | 6.48 | 538.5206 | [M+H]+ |
| **Cer(d36:1)** | 600.5129 | [M+Cl]- | 7.17 | 566.5513 | [M+H]+ |
| **Cer(d42:3)** | 680.573 | [M+Cl]- | 7.76 | 646.6104 | [M+H]+ |
| **Cer(d42:2)** | 682.5896 | [M+Cl]- | 8.39 | 648.6294 | [M+H]+ |
| **SM(d32:1)** | 709.5047 | [M+Cl]- | 4.98 | 675.5441 | [M+H]+ |
| **SM(d34:1)** | 737.5357 | [M+Cl]- | 5.68 | 703.5729 | [M+H]+ |
| **SM(d36:2)** | 763.549 | [M+Cl]- | 5.85 | 729.5834 | [M+H]+ |
| **SM(d40:1)** | 821.6258 | [M+Cl]- | 7.74 | 787.6468 | [M+H]+ |
| **SM(d42:2)** | 847.6445 | [M+Cl]- | 7.72 | 813.6838 | [M+H]+ |
| **SM(d42:1)** | 849.6547 | [M+Cl]- | 8.35 | 815.6997 | [M+H]+ |
| **SM(d44:2)** | 875.6753 | [M+Cl]- | 8.28 | 841.7149 | [M+H]+ |
| **TG(48:1)** | 839.6894 | [M+Cl]- | 10.44 | 822.7539 | [M+NH4]+ |
| **TG(50:9)** | 851.5939 | [M+Cl]- | 8.54 | 834.6795 | [M+NH4]+ |
| **TG(50:8)** | 853.611 | [M+Cl]- | 8.69 | 836.6904 | [M+NH4]+ |
| **TG(50:1)** | 867.7239 | [M+Cl]- | 10.76 | 850.7858 | [M+NH4]+ |

| | | | | | |
|---|---|---|---|---|---|
| **TG(52:9)** | 879.6294 | [M+Cl]- | 9.02 | 862.7018 | [M+NH4]+ |
| **TG(52:3)** | 891.7143 | [M+Cl]- | 10.53 | 874.7866 | [M+NH4]+ |
| **TG(52:2)** | 893.7352 | [M+Cl]- | 10.79 | 876.8014 | [M+NH4]+ |
| **TG(52:1)** | 895.7544 | [M+Cl]- | 11.03 | 878.817 | [M+NH4]+ |
| **TG(54:4)** | 917.7348 | [M+Cl]- | 10.58 | 900.7968 | [M+NH4]+ |
| **TG(54:3)** | 919.746 | [M+Cl]- | 10.82 | 902.8164 | [M+NH4]+ |
| **TG(54:2)** | 921.7629 | [M+Cl]- | 11.05 | 904.8322 | [M+NH4]+ |
| **TG(54:1)** | 923.7803 | [M+Cl]- | 11.28 | 906.8451 | [M+NH4]+ |
| **TG(56:7)** | 939.7181 | [M+Cl]- | 10.3 | 922.7836 | [M+NH4]+ |
| **TG(56:4)** | 945.7663 | [M+Cl]- | 10.86 | 928.8311 | [M+NH4]+ |
| **PE(O-32:2)** | 672.497 | [M-H]- | 6.42 | 672.4957 | [M-H]- |
| **PE(O-34:3)** | 698.5106 | [M-H]- | 6.56 | 698.5104 | [M-H]- |
| **PE(O-34:2)** | 700.5272 | [M-H]- | 7.07 | 700.5271 | [M-H]- |
| **PE(O-34:1)** | 702.5398 | [M-H]- | 7.57 | 702.5432 | [M-H]- |
| **PE(O-36:6)** | 720.4954 | [M-H]- | 5.01 | 720.4877 | [M-H]- |
| **PE(O-36:5)** | 722.5128 | [M-H]- | 6.52 | 722.5116 | [M-H]- |
| **PE(O-36:2)** | 728.559 | [M-H]- | 7.7 | 728.5589 | [M-H]- |
| **PE(O-38:7)** | 746.5113 | [M-H]- | 6.33 | 746.5211 | [M-H]- |
| **PE(O-38:6)** | 748.525 | [M-H]- | 6.56 | 748.5272 | [M-H]- |
| **PE(O-38:5)** | 750.5413 | [M-H]- | 7.19 | 750.5426 | [M-H]- |
| **PE(O-40:8)** | 772.5266 | [M-H]- | 6.43 | 772.5417 | [M-H]- |
| **PE(O-40:6)** | 776.5572 | [M-H]- | 7.16 | 776.5602 | [M-H]- |
| **PE(O-40:5)** | 778.5705 | [M-H]- | 7.58 | 778.5739 | [M-H]- |

## Table S3

Table with the amino acids validated and quantified with LC-MS/MS and standard compounds.

| REIMS m/z | ID |
|---|---|
| **146.0452** | Glutamate |
| **145.0613** | Glutamine |
| **154.0618** | Histidine |
| **148.0446** | Methionine |
| **164.0711** | Phenylalanine |
| **114.0535** | Proline |
| **132.0293** | Aspartate |
| **104.0348** | Serine |
| **118.0506** | Threonine |
| **203.083** | Tryptophan |
| **116.0693** | Valine |
| **124.0073** | Taurine |
